# Microwave-assisted efficient one-pot synthesis of *N*^2^-(tetrazol-5-yl)-6-aryl/heteroaryl-5,6-dihydro-1,3,5-triazine-2,4-diamines

**DOI:** 10.3762/bjoc.16.142

**Published:** 2020-07-16

**Authors:** Moustafa Sherief Moustafa, Ramadan Ahmed Mekheimer, Saleh Mohammed Al-Mousawi, Mohamed Abd-Elmonem, Hesham El-Zorba, Afaf Mohamed Abdel Hameed, Tahany Mahmoud Mohamed, Kamal Usef Sadek

**Affiliations:** 1 Department of Chemistry, Faculty of Science, Kuwait University, P.O. Box 12613, Safat,13060, Kuwait; 2 Department of Chemistry, Faculty of Science, Minia University, Minia 61519, Egypt; 3 Department of Pharmacology, Faculty of Veterinary Medicine, Cairo University, Giza 12211, Egypt

**Keywords:** microwave irradiation, *N*^2^-(tetrazol-5-yl)-6-aryl/heteroaryl-1,3,5-triazine-2,4-diamines, one-pot synthesis, X-ray crystallography

## Abstract

An efficient one-pot synthesis of *N*^2^-(tetrazol-5-yl)-6-aryl/heteroaryl-1,3,5-triazine-2,4-diamine derivatives was developed by reacting 5-amino-1,2,3,4-tetrazole with aromatic aldehydes and cyanamide in pyridine under controlled microwave heating with high yields. X-ray crystallography confirmed the structure of the obtained products.

## Introduction

The family of triazines is of considerable interest in fields related to organic and medicinal chemistry. 2,4-Diaminotriazines are privileged scaffolds exhibiting diverse biological activities such as antibacterial [1], anti-HSV-1 [2], antitumor [3], anti-HIV [4], inhibitor of *Trypanosoma brucei* [5], angiogenesis inhibitor [6], antiplasmodial antifolates [7], and antimicrobial [8]. Moreover, and in particular *N*^2^,6-disubstituted-1,3,5-triazine-2,4-diamines possess a wide range of chemotherapeutic activities [8–11].

Tetrazole derivatives are a potent class of heterocyclic compounds with a wide range of biological activities owing to their unique structure. They play an important role not only as a bioisostere of the carboxylic acid group but also as flexible ligands which easily adopt to different binding modes [12–13]. Tetrazole derivatives exhibit a wide spectrum of biological activities as antibacterial [14], anticancer [15], anti-inflammatory [16], antidiabetic [17], antitubercular [18], and analgesic [19] agents.

It is well established that many medical disorders can be caused as a result of defects at more than one specific biological target such as a receptor or an enzyme. A promising strategy that overcomes the classical one-target, one-molecule approach is the design of stable chemical hybrid molecules which are a combination of two biologically active scaffolds acting at different targets [20–24]. Accordingly, we reasoned that heterocycles incorporating both an *N*^2^-(tetrazol-5-yl) ring system and a 1,3,5-triazine-2,4-diamine scaffold could be very effective biologically relevant heterocycles.

**Scheme 1:**
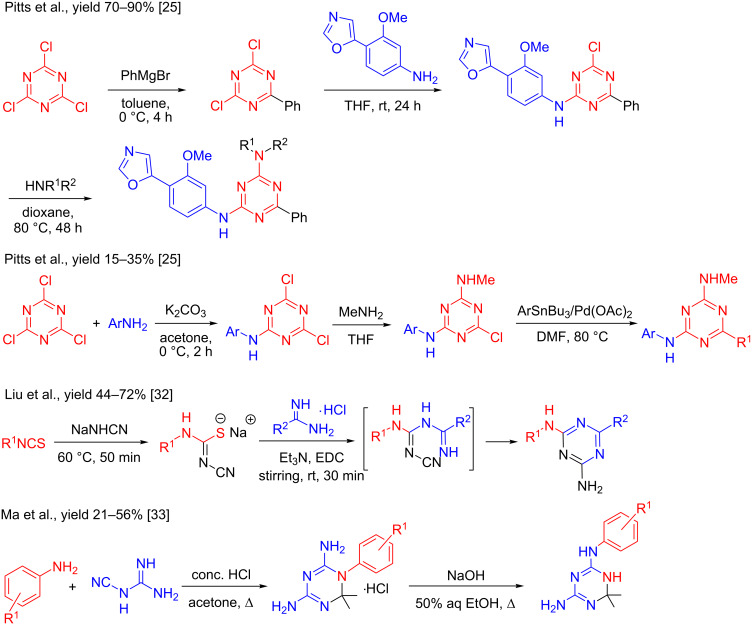
Previously reported methods for the synthesis of 1,3,5-triazine-2,4-diamine derivatives.

Little attention has been paid to the synthesis of *N*^2^,6-disubstituted-1,3,5-triazine-2,4-diamines which requires a multistep synthesis route. A first approach relied on the nucleophilic substitution of chlorine in cyanuric chloride with Grignard reagents, ammonia or amines [25–26], which suffered from the high reactivity of the Grignard reagents that prevents further functionalization. Moreover, this protocol required temperature control and showed dependence on the amine nucleophile reactivity [27]. Another route involved the reaction of substituted biguanidines with acetic anhydrides, chlorides or carboxylates [11,28–31]. Liu et al. [32] reported a one-pot synthesis of *N*^2^,6-disubstituted-1,3,5-triazine-2,4-diamines in 44–72% yields that employed the reaction of isothiocyanates with sodium hydrogen cyanamide and amidines in the presence of 1-(3-dimethylaminopropyl)-3-ethylcarbodiimide hydrochloride and heating at 75 °C for 3 h. Recently, Ma et al. [33] described a one-pot two step procedure for the synthesis of 6-substituted-*N*^2^-aryl-1,6-dihydro-1,3,5-triazine-2,4-diamines via the reaction of aromatic amines, cyanoguanidine, and ketones which afforded the corresponding 1-aryl-1,6-dihydro-6-substituted-1,3,5-triazine-2,4-diamines in 21–56% yields followed by Dimroth rearrangement utilizing sodium hydroxide (50%) in aqueous ethanol (Scheme 1).

Although, these methods have specific merits, they sometimes suffer from drawbacks such as extended reaction temperatures, lengthy procedures, low yields and atom economy, which consume excess reagents. Extensive efforts have been devoted to adopting green methodologies in synthetic heterocyclic chemistry. The utilization of microwave heating as an energy source has several advantages including operational simplicity, high reaction yields, enhanced rates, and increased energy efficiency [34–40].

**Scheme 2:**
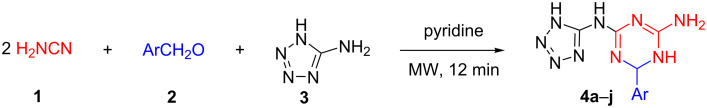
One-pot synthesis of *N*^2^-(tetrazol-5-yl)-6-aryl/heteroaryl-5,6-dihydro-1,3,5-triazine-2,4-diamines **4a**–**j**.

In continuation of our efforts in performing green methodologies in the synthesis of biologically relevant heterocycles from simple starting materials [41–44], we developed an efficient synthesis of *N*^2^-(tetrazol-5-yl)-6-aryl/heteroaryl-1,3,5-triazine-2,4-diamines through the one-pot reaction of cyanamide **1**, aromatic aldehydes **2**, and 5-aminotetrazole (**3**) in pyridine under controlled microwave heating (Scheme 2).

## Results and Discussion

With the initial aim of optimizing the reaction conditions, we began our study by reacting equimolar amounts of cyanamide **1**, aromatic aldehydes **2**, and 5-aminotetrazole (**3**) in pyridine and the reaction was promoted by microwave heating at 120 °C over 12 min. After cooling the reaction mixture to room temperature and work-up, a solid product was obtained in low yield (40%) and confirmed to be 6-(4-chlorophenyl)-*N*^2^-(1*H*-tetrazol-5-yl)-5,6-dihydro-1,3,5-triazine-2,4-diamine (**4a**) based on analytical and spectral data. The mass spectrum of the reaction product showed a molecular ion peak at *m*/*z* = 290.1 [M − 1]^+^. The ^1^H NMR revealed four singlet signals at δ = 11.22, 10.81, 8.72, and 6.17 ppm each integrated for one proton which were assigned to the triazine-NH, NH at *N*^2^-(tetrazole-5-yl), tetrazole NH, and triazine CH-2 protons in addition to two broad singlet signals at δ = 8.59 and 7.32 ppm for NH_2_ function as well as signals for the aromatic protons. The ^13^C NMR spectrum was in support of the proposed structure. Based on the established product, we revealed that two molecules of cyanamide **1** participated in the reaction course and the yield was increased to 92% when the molar ratio of the reactants **1**, **2**, and **3** was set at 2:1:1. We next surveyed a structurally diverse group of aromatic aldehydes **2** with cyanamide **1** and 5-aminotetrazole (**3**) under the same experimental conditions and the results are summarized in (Table 1 and Scheme 2). Irrespective of the aryl group either electron-donating or electron-withdrawing, the reaction proceeded smoothly and gave a variety of 1,3,5-triazine-2,4-diamines **4** in high yields.

**Table 1:** Microwave three-component synthesis of triazines **4a**–**j**.

Entry	Ar	Product	Yield (%)

1	4-ClC_6_H_4_	**4a**	92
2	4-OMeC_6_H_4_	**4b**	89
3	C_6_H_5_	**4c**	93
4	benzo[*d*]dioxol	**4d**	88
5	2-OMeC_6_H_4_	**4e**	88
6	4-MeC_6_H_4_	**4f**	87
7	2-ClC_6_H_4_	**4g**	92
8	3-NO_2_C_6_H_4_	**4h**	93
9	2-furyl	**4i**	91
10	4-NMe_2_C_6_H_4_	**4j**	88

The effect of the solvent was also examined. Other solvents were screened under the same experimental conditions and the results revealed that performing the reaction in dioxane, CH_3_CN, THF, or catalyst-free ethanol resulted in no product formation. However, the same products were obtained with lower yields (≈60%) when performing the reaction under conventional heating utilizing pyridine as the solvent for 3 hours. These results demonstrated the advantage of microwave heating as an efficient energy source.

**Figure 1:**
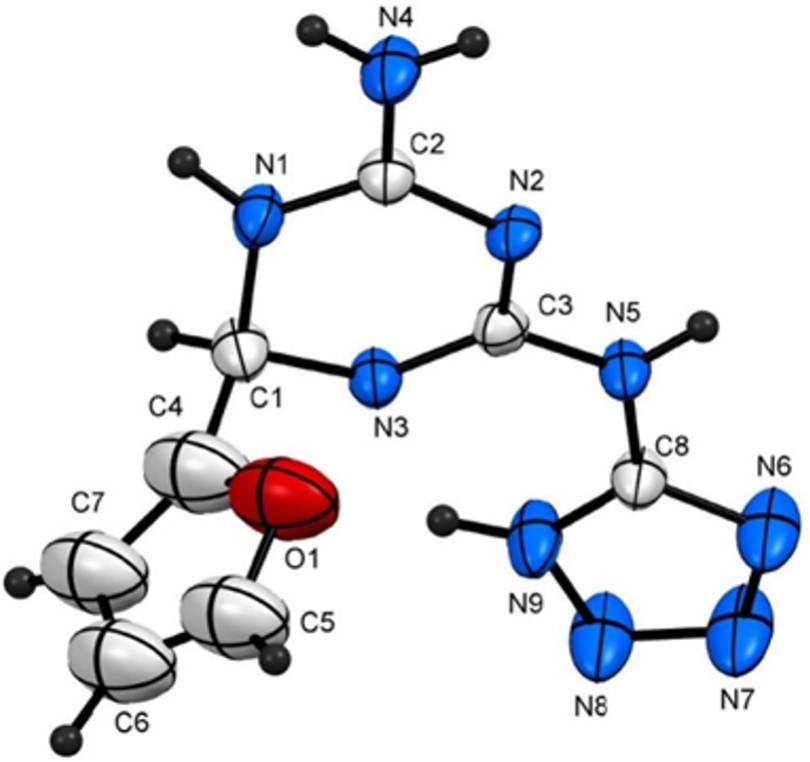
ORTEP diagram of compound **4i**.

**Scheme 3:**
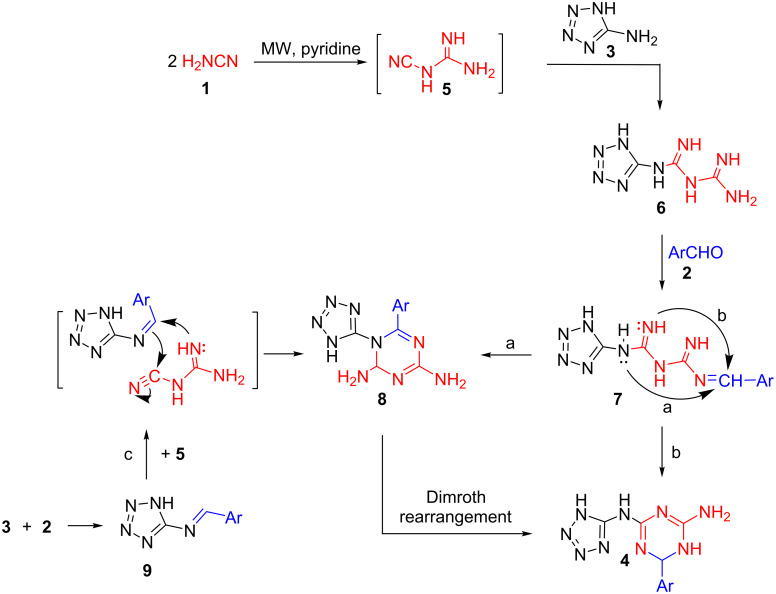
Plausible different routes to account for the formation of products **4**.

The structure proposed for the reaction products was established on the bases of analytical and spectral data (MS, ^1^H NMR, ^13^C NMR, and elemental analyses). Moreover, the structure of **4i** was unequivocally supported by single-crystal X-ray diffraction (Figure 1 and Table 2). A plausible mechanism for the formation of products **4** is postulated in Scheme 3.

**Table 2:** Selected bond lengths and bond angles for compound **4i**.

bond lengths		bond angles	
atom numbers	geometric parameter (A°)	atom numbers	geometric parameter (°)

			
O1–C5	1.360 (9)	C1–N1–C2	121.6 (4)
C4–C7	1.326 (11)	C2–N2–C3	115.0 (4)
C1–C4	1.465 (9)	N1–C2–N4	118.2 (4)
N6–N7	1.336 (7)	C1–C4–C7	136.2 (8)
N5–N3	1.339 (6)	C4–O1–C5	109.8 (6)
N1–C1	1.453 (6)	N6–N7–N8	109.9 (5)
C1–C4	1.465 (12)	N8–N9–C8	104.4 (4)
N3–C1	1.459 (6)	N2–C3–N5	116.1 (4)
		O1–C4–C1	116.6 (6)
		O1–C5–C6	106.2 (7)

The dimerization of cyanamide **1** in basic medium to cyanoguanidine **5** and subsequent reaction with 5-aminotetrazole (**3**) yielded tetrazolylbiguanidine **6** which undergoes a condensation reaction with aromatic aldehydes **2** to afford **7**. The nucleophilic attack of the secondary amine in **7** to the arylidene carbon gives rise to the formation of 6-aryl-1-(1*H*-tetrazol-5-yl)-1,2-dihydro-1,3,5-triazine-2,4-diamine intermediate **8** (route a) or the nucleophilic attack of the primary amine in **7** to the same imine carbon produces the corresponding *N*^2^-(tetrazole-5-yl)-6-aryl-1,3,5-triazine-2,4-diamines **4** (route b). Alternatively, intermediate **8** could be obtained by a condensation of 5-aminotetrazole (**3**) with aromatic aldehydes **2** followed by the addition of cyanoguanidine **5** to the formed Schiff’s base (route c). Product **4** was the sole isolable product as under reflux in pyridine as base, compound **8** well undergoes a Dimroth rearrangement forming the more thermodynamically stable product **4** [33]. We established route c, as the formation of the Schiff base is more favorable due to the high nucleophilicity of the exocyclic amino function attached to the electron-rich tetrazole ring [45]. In support of this assumption we stopped the reaction after 4 minutes of heating under microwave irradiation and inspected the prior formation of **9** via comparison with an authentic sample synthesized by a conventional method.

## Conclusion

The synthesis of biologically relevant 6-aryl/heteroaryl-*N*^2^-(5*H*-tetrazole-5-yl)-5,6-dihydro-1,3,5-triazine-2,4-diamines was achieved under controlled microwave heating via a simple one-pot, three-component reaction of cyanamide **1**, aldehydes **2**, and 5-amino-1,2,3,4-tetrazole (**3**) in excellent yields. The process proved to be an efficient synthetic route displaying high atom economy, short reaction times, and a simple work-up procedure. This protocol appeared to be general with a diversity of amines and aldehydes.

## Experimental

All chemicals were purchased from Aldrich or Merck Companies. The ^1^H NMR (600 MHz) and ^13^C NMR (150 MHz) were run in a Bruker DPX instrument (δ ppm). Mass spectra were measured by using VG Autospec Q MS 30 and MS 9 (AEI) spectrometer, with EI (70 eV) mode. Melting points were recorded in a Gallenkamp melting point apparatus and are uncorrected. X-ray crystallographic structure determinations were performed by using Rigaku Rapid II and Bruker X8 Prospector single crystal X-ray diffractometers. The X-ray crystal structure data can be obtained free of charge from the Cambridge Crystallographic Data Centre via http://www.ccdc.cam.ac.uk CCDC1961565 for compound **4i**. All reactions were monitored by TLC with 1:1 ethyl acetate/petroleum ether as eluent and were carried out until starting materials were completely consumed. After 7 min microwave irradiation was stopped and the reaction mixture was analyzed by TLC; after further irradiation of 5 min the reaction was complete (total reaction time 12 min).

### General procedure for the synthesis of *N*^2^,6-disubstituted dihydro-1,3,5-triazine-2,4-diamine derivatives

A solution of **1** (2 mmol), **2** (1 mmol), and **3** (1 mmol) in pyridine (10 mL) was heated under reflux in a Milestone Microwave Labstation at 120 °C for 12 min. The solvent was removed under reduced pressure and the solid product was isolated by filtration and recrystallized from DMF.

**6-(4-Chlorophenyl)-*N*^2^-(5*H*-tetrazol-5-yl)-5,6-dihydro-1,3,5-triazine-2,4-diamine (4a).** Colorless crystals; mp 320–322 °C; yield 0.268 g, 92%; *R*_f_ 0.55 (1:1 ethyl acetate/petroleum ether); ^1^H NMR (600 MHz, DMSO-*d*_6_) δ 6.17 (s, 1H), 7.32 (s, 1H), 7.46 (d, *J* = 8.4 Hz, 2H), 7.53 (d, *J* = 9.2 Hz, 2H), 8.58 (s, 1H), 8.73 (brs, 1H), 10.82 (s, 1H), 11.22 (s, 1H); ^13^C NMR (150 MHz, DMSO-*d*_6_) δ 61.52, 127.84, 128.94, 133.70, 139.75, 154.45, 157.61, 158.0; anal. calcd for C_10_H_10_ClN_9_: C, 41.17; H, 3.46; Cl, 12.15; N, 43.22; found: C, 41.22; H, 3.42; Cl, 12.11; N, 43.24; EIMS (*m*/*z*): 290.1 [M − 1]^+^.

**6-(4-Methoxyphenyl)-*N*^2^-(5*H*-tetrazol-5-yl)-5,6-dihydro-1,3,5-triazine-2,4-diamine (4b).** Colorless crystals; mp 306–308 °C; yield 0.255 g, 89%; ^1^H NMR (600 MHz, DMSO-*d*_6_) δ 3.74 (s, 3H), 6.07 (s, 1H), 7.01 (d, *J* = 8.4, Hz, 2H), 7.38 (br s, 1H), 7.39 (d, *J* = 9 Hz, 2H), 8.29 (s, 1H), 8.60 (s, 1H), 10.69 (s, 1H), 11.15 (s, 1H); ^13^C NMR (150 MHz, DMSO-*d*_6_) δ 55.26, 62.01, 114.29, 127.51, 132.45, 154.57, 157.79, 158.14, 159.92; anal. calcd for C_11_H_13_N_9_O: C, 45.99; H, 4.56; N, 43.88; found: C, 45.89; H, 4.52; N, 43.90; EIMS (*m*/*z*): 286.1 [M − 1]^+^.

**6-Phenyl-*N*^2^-(5*H*-tetrazol-5-yl)-5,6-dihydro-1,3,5-triazine-2,4-diamine (4c).** Colorless crystals; mp 317–319 °C; yield 0.277 g, 93%; ^1^H NMR (600 MHz, DMSO-*d*_6_) δ 6.15 (s, 1H), 7.22 (s, 1H), 7.40–7.46 (m, 5H), 8.50 (br s, 1H), 8.71 (s, 1H), 10.77 (s, 1H), 11.20 (s, 1H); ^13^C NMR (150 MHz, DMSO-*d*_6_) δ 62.2, 125.9, 128.9, 129.1, 154.5, 157.7, 158.0; anal. calcd for C_10_H_11_N_9_: C, 46.69; H,4.31; N, 49.0; found: C, 46.76; H, 4.41; N, 48.85; EIMS (*m*/*z*): 256.1 [M − 1]^+^.

**6-(Benzo[*d*][1,3]dioxol-5-yl)-*N*^2^-(5*H*-tetrazol-5-yl)-5,6-dihydro-1,3,5-triazine-2,4-diamine (4d).** Colorless crystals; mp 304–306 °C; yield 0.265 g, 88%; ^1^H NMR (600 MHz, DMSO-*d*_6_) δ, 6.02 (s, 1H), 6.04 (d, 2H), 6.90–7.12 (m, 4H), 8.02 (brs, 1H), 8.55 (s, 1H), 10.68 (s, 1H), 11.10 (s, 1H); ^13^C NMR (150 MHz, DMSO-*d*_6_) δ 56.0, 62.0, 101.4, 106.3, 108.29, 119.63, 134.38, 147.76, 147.93, 154.49, 157.72, 158.15; anal. calcd for C_11_H_11_N_9_O_2_: C, 43.85; H, 3.68; N, 41.84; found: C, 43.76; H, 3.59, N, 41.79.

**6-(2-Methoxyphenyl)-*N*^2^-(5*H*-tetrazol-5-yl)-5,6-dihydro-1,3,5-triazine-2,4-diamine (4e).** Colorless crystals; mp 310–312 °C; yield 0.247 g, 88%; ^1^H NMR (600 MHz, DMSO-*d*_6_) δ 3.89 (s, 3H), 6.27 (s, 1H), 7.02 (t, *J* = 8.4 Hz, 1H), 7.12 (d, *J* = 8.4 Hz, 1H), 7.20, 7.21 (dd, *J* = 8.4, 1.8 Hz, 1H), 7.38–7.41 (m, 1H), 8.04 (brs, 1H), 8.36 (s, 1H), 10.87 (s, 1H), 11.07 (s, 1H); ^13^C NMR (150 MHz, DMSO-*d*_6_) δ 55.85, 58.74, 111.65, 120.42, 125.28, 127.77, 130.50, 154.61, 156.3, 157.97, 158.2; anal. calcd for C_11_H_13_N_9_O: C, 45.99; H, 4.56; N, 43.88; found: C, 46.10; H, 4.69; N, 43.81; EIMS (*m*/*z*): 286.1 [M − 1]^+^.

**6-(4-Methylphenyl)-*N*^2^-(5*H*-tetrazol-5-yl)-5,6-dihydro-1,3,5-triazine-2,4-diamine (4f).** Colorless crystals; mp 314−316 °C; yield 0.235 g, 87%; ^1^H NMR (600 MHz, DMSO-*d*_6_) δ 2.50 (s, 3H), 6.09 (s, 1H), 7.19 (s, 1H), 7.26 (d, *J* = 8.4 Hz, 2H), 7.34 (d, *J* = 8.4 Hz, 2H), 8.47 (brs, 1H), 8.66 (s, 1H), 10.7 (s, 1H), 11.18 (s, 1H); ^13^C NMR (150 MHz, DMSO-*d*_6_) δ 20.73, 62.08, 125.90, 129.41, 137.64, 138.69, 154.53, 157.76, 158.07; anal. calcd for C_11_H_13_N_9_: C, 48.70; H, 4.83; N, 46.47; found: C, 48.75; H, 4.70; N, 46.56.

**6-(2-Chlorophenyl)-*N*^2^-(5*H*-tetrazol-5-yl)-5,6-dihydro-1,3,5-triazine-2,4-diamine (4g).** Colorless crystals; mp 324–326 °C; yield 0.267 g, 92%; ^1^H NMR (600 MHz, DMSO-*d*_6_) δ 6.44 (s, 1H), 7.22 (s, 1H), 7.42–7.44 (m, 1H), 7.45–7.48 (m, 2H), 7.56–7.59 (m, 1H), 8.55 (brs, 1H), 8.65 (s, 1H), 10.94 (s, 1H), 11.29 (s, 1H); ^13^C NMR (150 MHz, DMSO-*d*_6_) δ 60.55, 127.39, 127.98, 130.30, 131.05, 131.42, 137.07, 154.52, 157.71, 158.04, 162.31; anal. calcd for C_10_H_10_ClN_9_: C, 41.17; H, 3.46; Cl, 12.15; N, 43.22; found: C, 41.30; H, 3.34; Cl, 12.30; N, 43.38.

**6-(3-Nitrophenyl)-*N*^2^-(5*H*-tetrazol-5-yl)-5,6-dihydro-1,3,5-triazine-2,4-diamine (4h).** Colorless crystals; mp 298–300 °C; yield 0.281 g, 93%; ^1^H NMR (600 MHz, DMSO-*d*_6_) δ 6.35 (s, 1H), 7.52 (s, 1H), 7.76 (t, *J* = 7.2 Hz, 1H), 7.88 (s, 1H), 8.26 (d, *J* = 7.8 Hz, 1H), 8.34 (s, 1H), 8.88 (brs, 2H), 10.95 (s, 1H), 11.26 (s, 1H); ^13^C NMR (150 MHz, DMSO-*d*_6_) δ 61.28, 120.92, 123.91, 130.73, 132.27, 143.13, 147.95, 154.42, 157.62, 157.94; anal. calcd for C_10_H_10_N_10_O_2_: C, 39.74; H, 3.33; N, 46.34; found: C, 39.68; H, 3.47; N, 46.52.

**6-(Furan-2-yl)-*N*^2^-(5*H*-tetrazol-5-yl)-5,6-dihydro-1,3,5-triazine-2,4-diamine (4i).** Colorless crystals; mp 208–210 °C; yield 0.224 g, 91%; ^1^H NMR (600 MHz, DMSO-*d*_6_) δ 6.24 (s, 1H), 6.46 (d, *J* = 16.8 Hz, 2H), 7.29 (brs, 1H), 7.70 (s, 1H), 8.52 (brs, 1H), 8.69 (s, 1H), 10.76 (s, 1H), 11.21 (s, 1H); ^13^C NMR (150 MHz, DMSO-*d*_6_) δ 56.48, 107.88, 110.61, 143.88, 152.35, 154.52, 157.68, 157.97; anal. calcd for C_8_H_9_N_9_O: C, 38.87; H, 3.67; N, 50.99; found: C, 38.68; H, 3.76; N, 51.06; EIMS (*m*/*z*): 246.1 [M − 1]^+^.

**6-(4-*N,N*-Dimethylaminophenyl)-*N*^2^-(5*H*-tetrazol-5-yl)-5,6-dihydro-1,3,5-triazine-2,4-diamine (4j)**. Colorless crystals; mp 278–280 °C; yield 0.264 g, 88%; ^1^H NMR (600 MHz, DMSO-*d*_6_) δ 2.91 (s, 6H), 5.96 (s, 1H), 6.59 (d, *J* = 8.4 Hz, 2H), 7.01 (s, 1H), 7.20 (d, *J* = 8.4 Hz, 2H), 7.78 (brs, 1H), 8.46 (s, 1H), 10.59 (s, 1H), 11.03 (s, 1H); ^13^C NMR (150 MHz, DMSO-*d*_6_) δ 39.97, 40.03, 62.36, 111.42, 112.15, 127.01, 127.17, 128.32, 151.04, 154.64, 157.90, 158.24; anal. calcd for C_12_H_16_N_10_: C, 47.99; H, 5.37; N, 46.64; found: C, 48.10; H, 5.43; N, 46.52.

## Supporting Information

File 1 NMR and mass spectra.
