# Supplementary material for: Microwave-assisted efficient one-pot synthesis of N2-(tetrazol-5-yl)-6-aryl/heteroaryl-5,6-dihydro-1,3,5-triazine-2,4-diamines
Source: Beilstein J Org Chem. 2020 Jul 16;16:1706–12. doi: 10.3762/bjoc.16.142 (PMC7372238; doi:10.3762/bjoc.16.142)

BEILSTEIN JOURNAL OF ORGANIC CHEMISTRY

Supporting Information

for

Microwave-assisted efficient one-pot synthesis of N^2-(tetrazol-5-yl)-6-aryl/heteroaryl-5,6-dihydro-1,3,5-triazine-2,4-diamines

Moustafa Sherief Moustafa, Ramadan Ahmed Mekheimer,
Saleh Mohammed Al-Mousawi, Mohamed Abd-Elmonem, Hesham El-Zorba,
Afaf Mohamed Abdel Hameed, Tahany Mahmoud Mohamed and Kamal Usef Sadek

Beilstein J. Org. Chem. **2020,** *16,* 1706–1712. doi:10.3762/bjoc.16.142

NMR and mass spectra

License and Terms: This is a supporting information file under the terms of the Creative Commons Attribution License (http://creativecommons.org/licenses/by/4.0). Please note that the reuse, redistribution and reproduction in particular requires that the authors and source are credited.
The license is subject to the *Beilstein Journal of Organic Chemistry* terms and conditions: (https://www.beilstein-journals.org/bjoc)

6-(4-Chlorophenyl)-*N*^{2}-(5*H*-tetrazol-5-yl)-5,6-dihydro-1,3,5-triazine-2,4-diamine (4a).

This compound is novel.

4a ^{1}H NMR spectrum (600 MHz, DMSO-d_6).

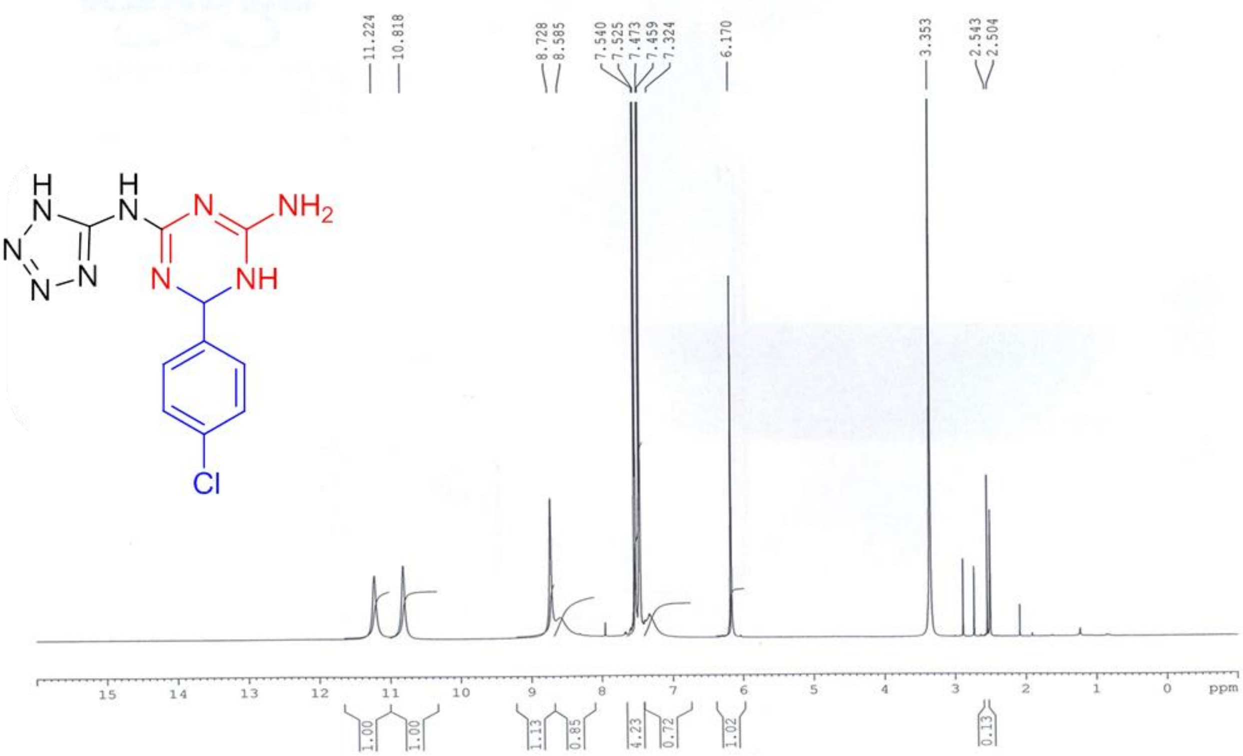

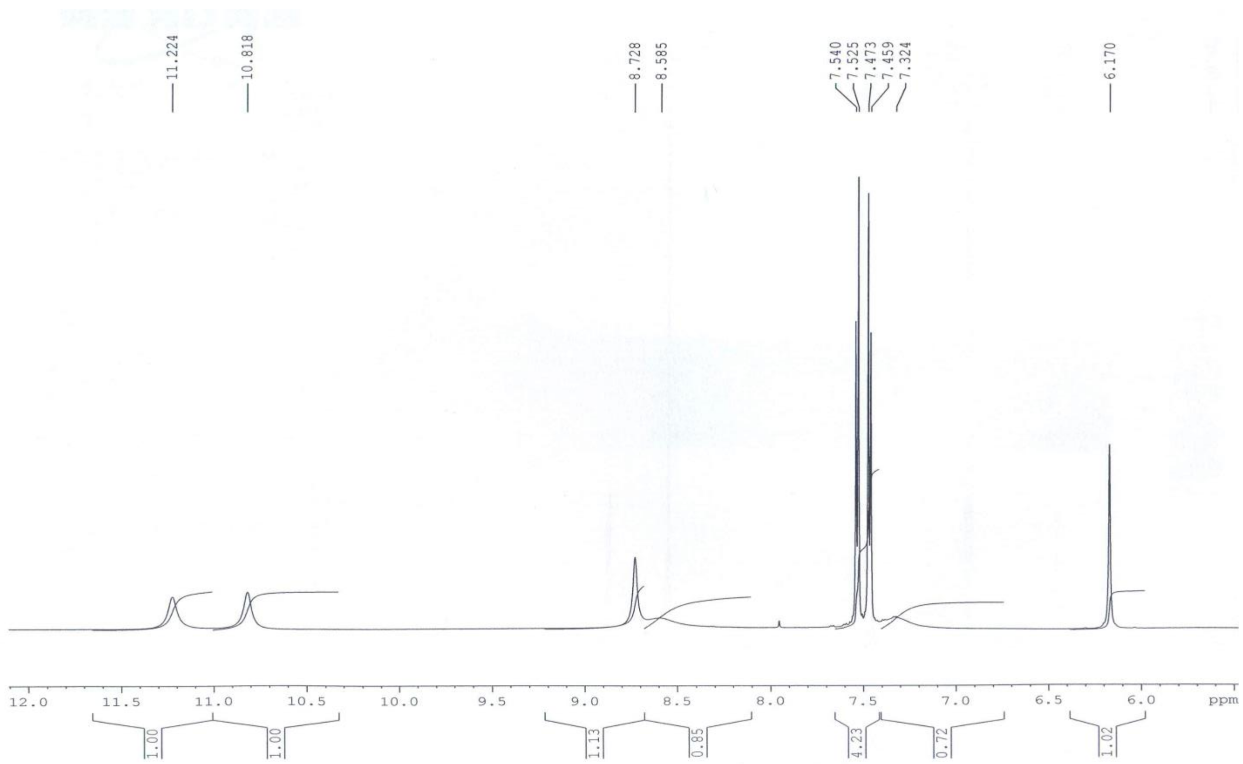

4a ^{13}C NMR spectrum (150 MHz, DMSO-d_6).

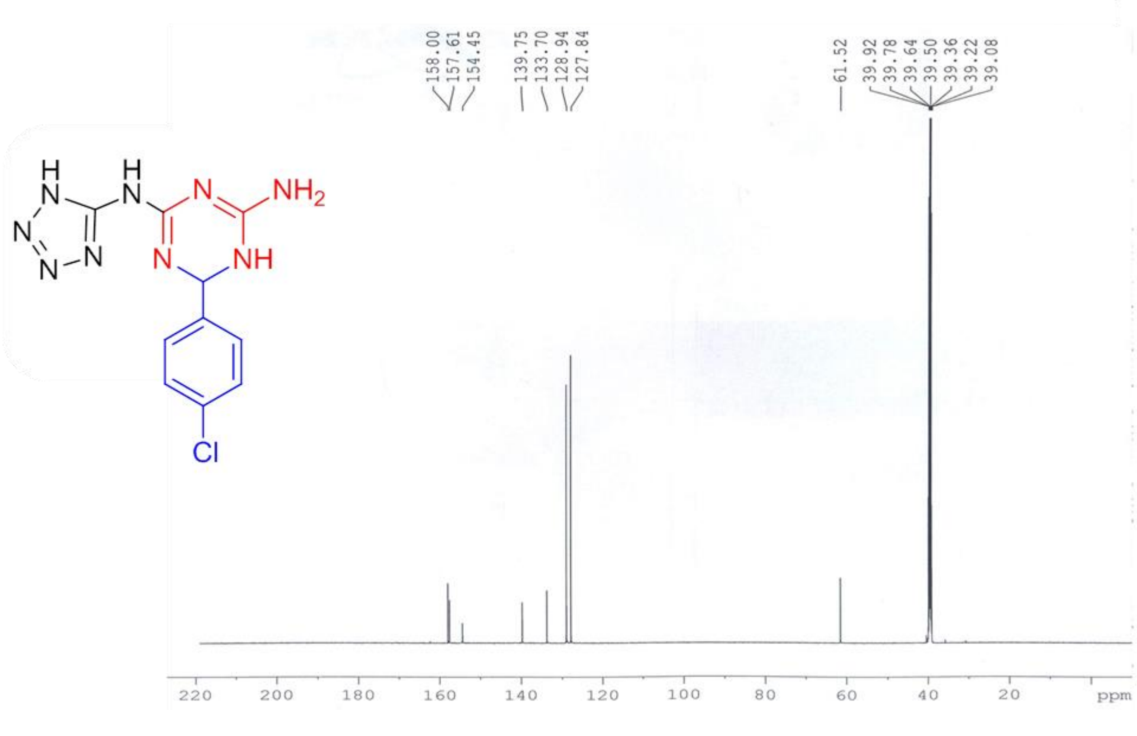

4a Mass spectrum.

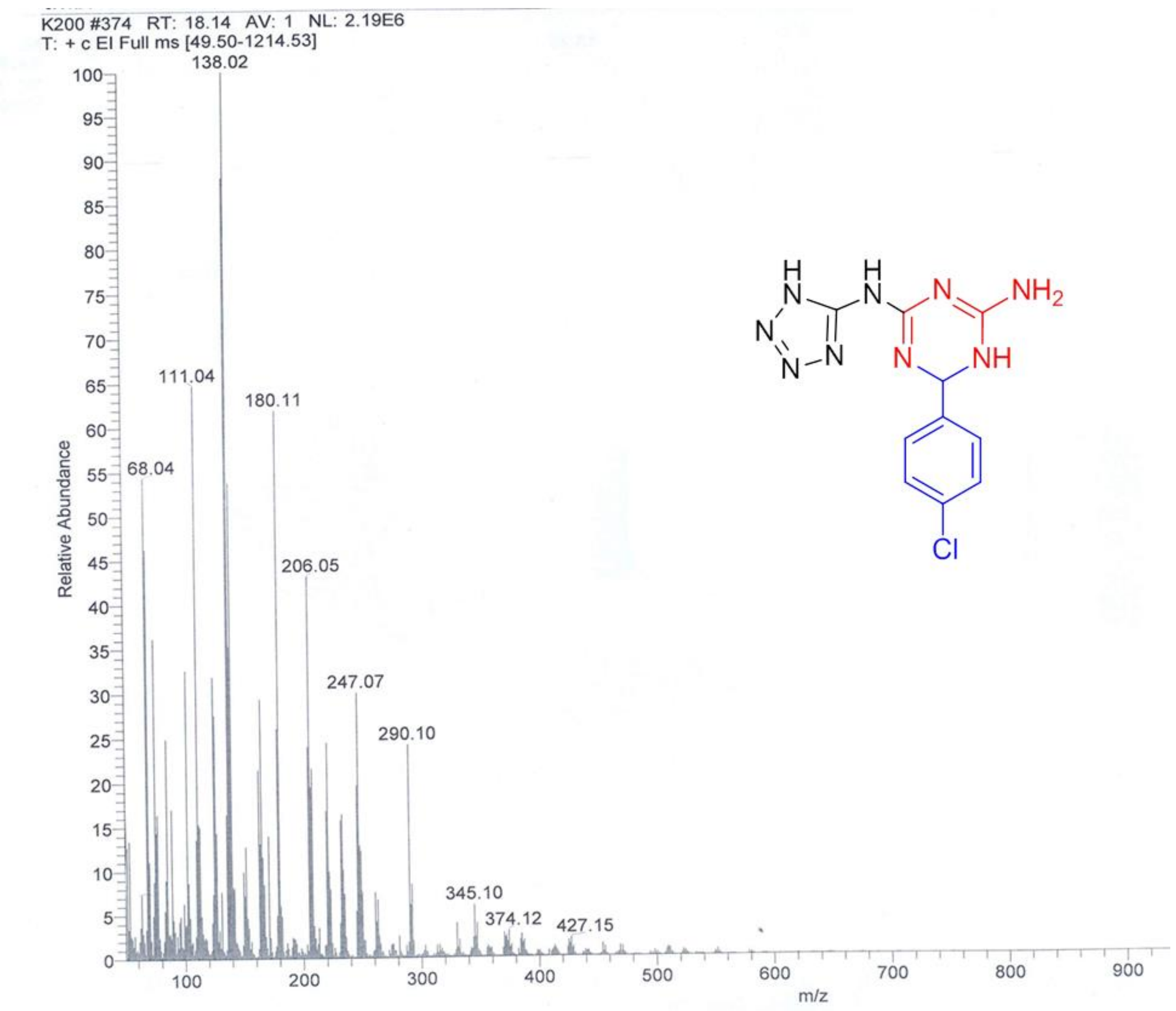

6-(4-Methoxyphenyl)-*N*²-(5*H*-tetrazol-5-yl)-5,6-dihydro-1,3,5-triazine-2,4-diamine (4b).

This compound is novel.

4b ^{1}H NMR spectrum (600 MHz, DMSO-d_6).

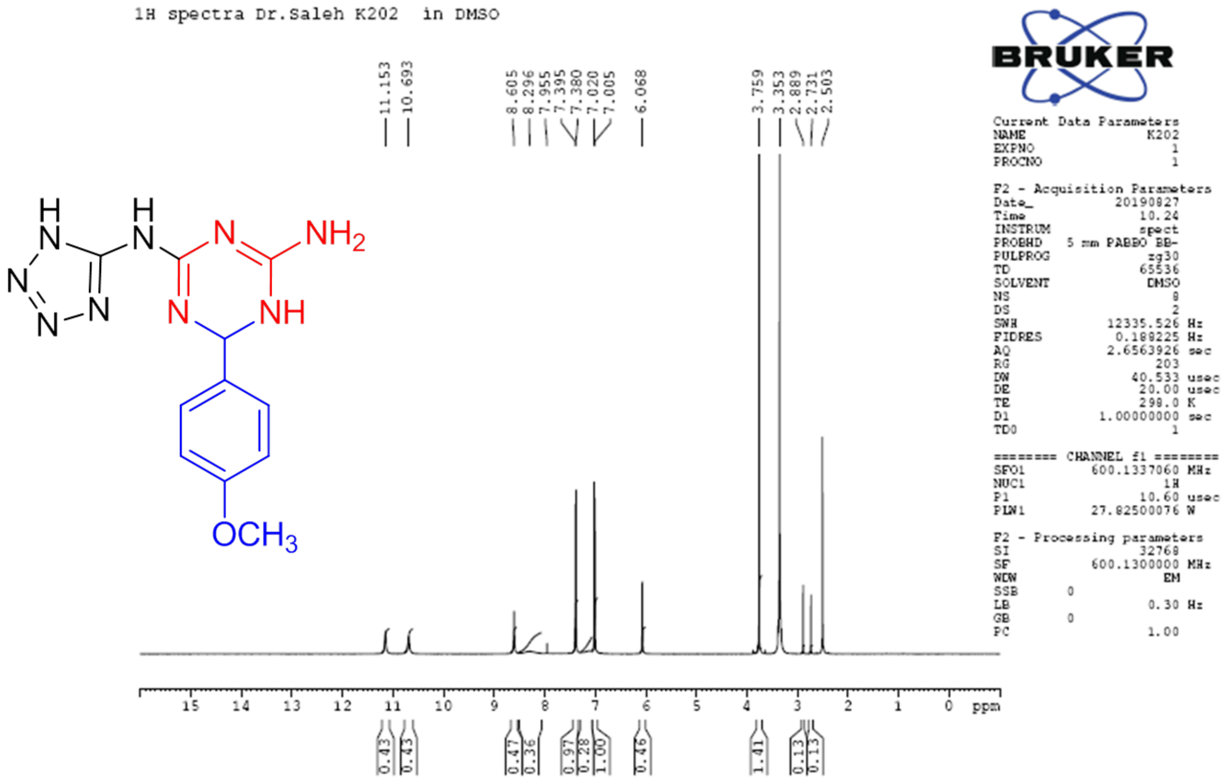

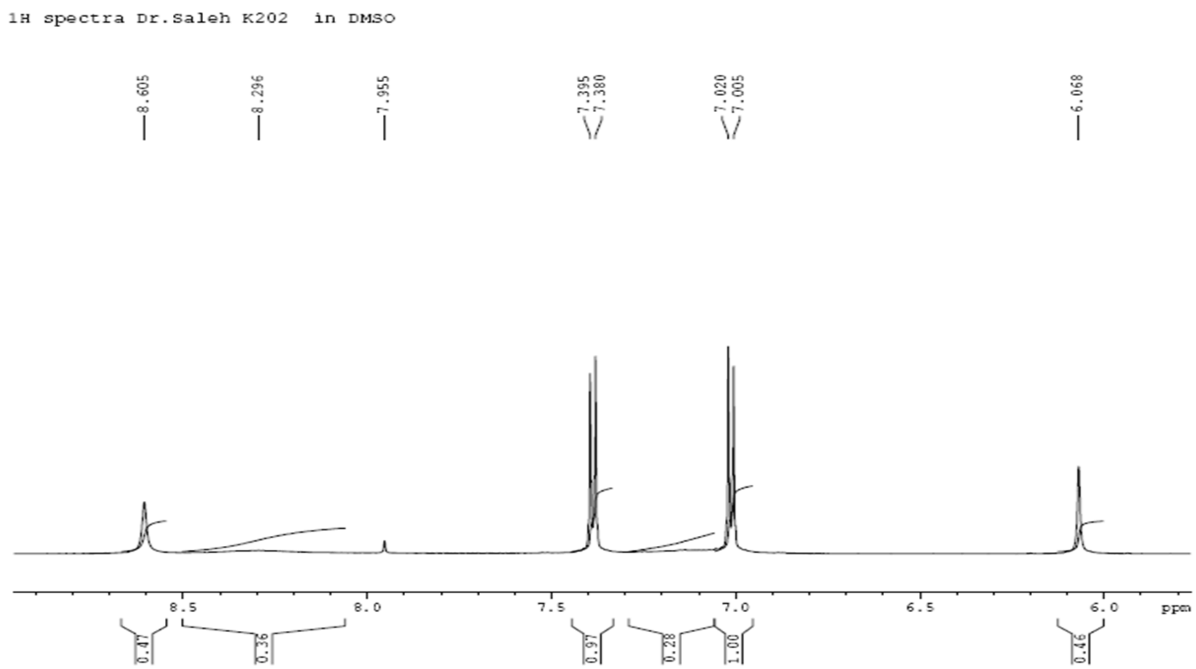

4b ^{13}C NMR spectrum (150 MHz, DMSO-d_6).

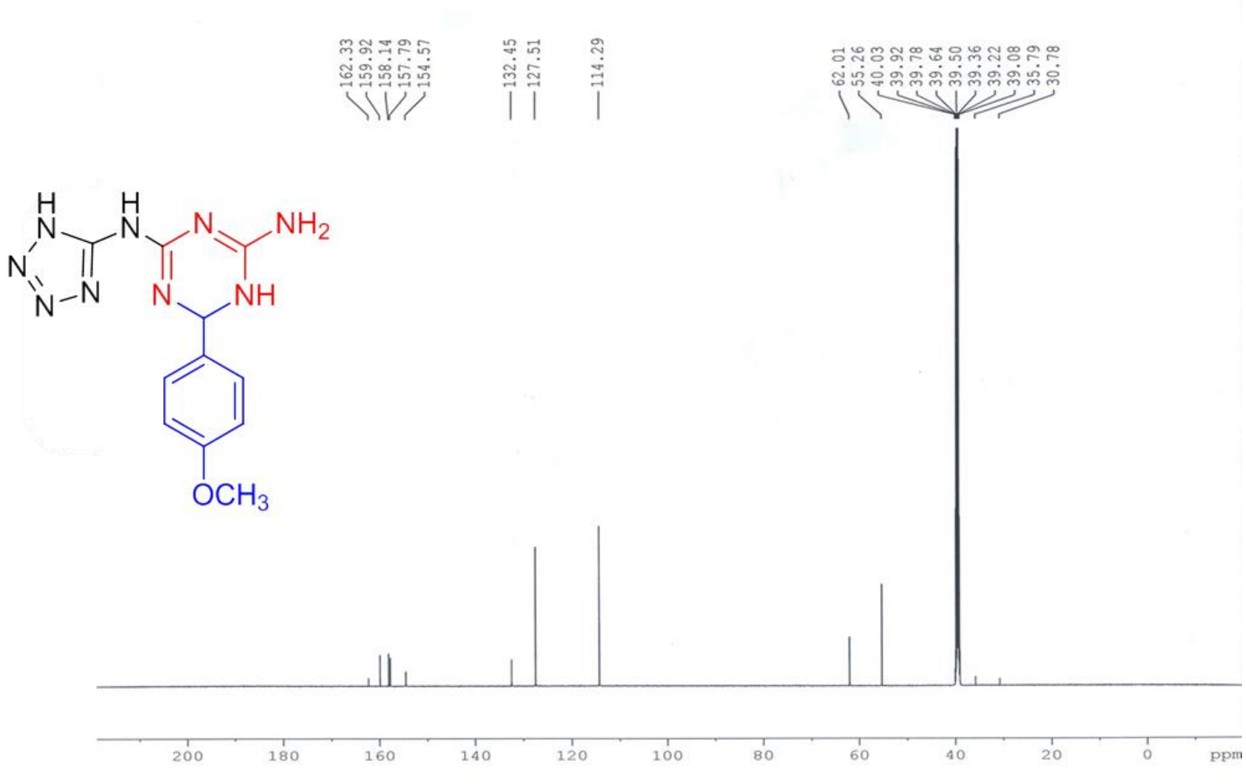

4b Mass spectrum.

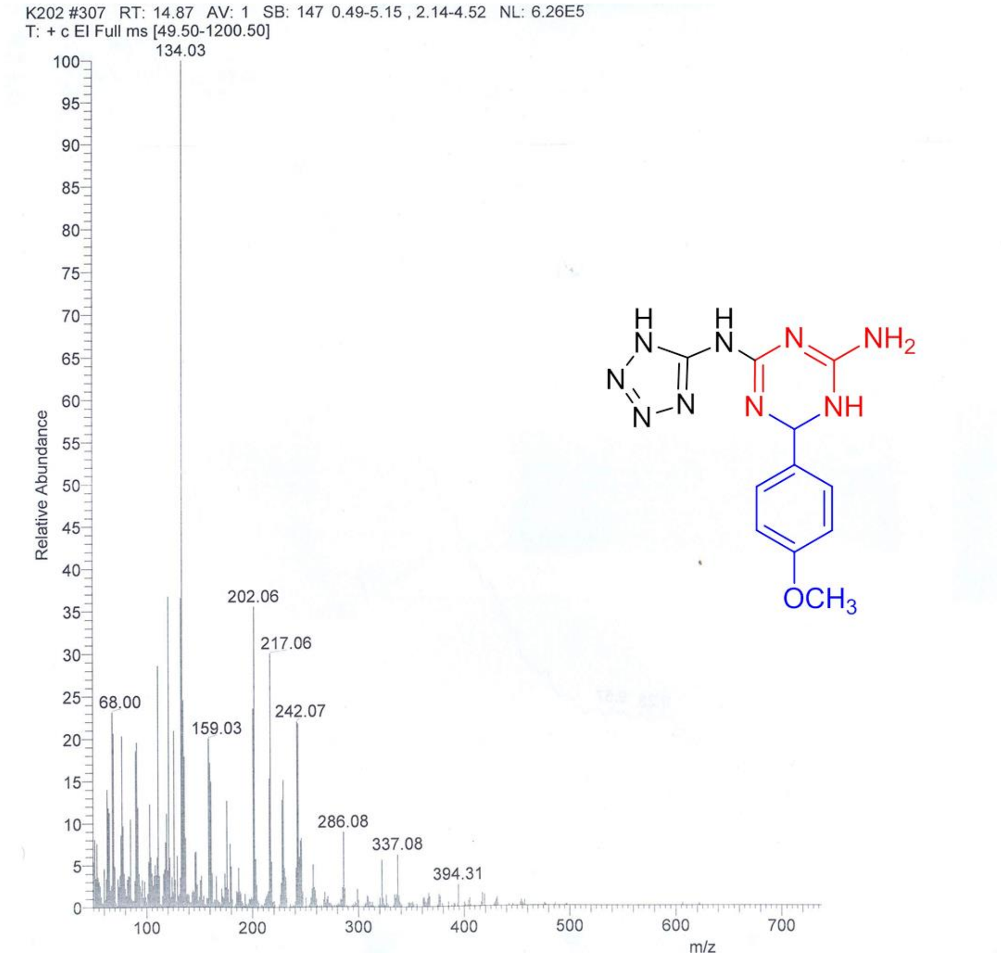

6-Phenyl-N^2-(5H-tetrazol-5-yl)-5,6-dihydro-1,3,5-triazine-2,4-diamine (4c).

This compound is novel.

4c ^{1}H NMR spectrum (600 MHz, DMSO-d_6).

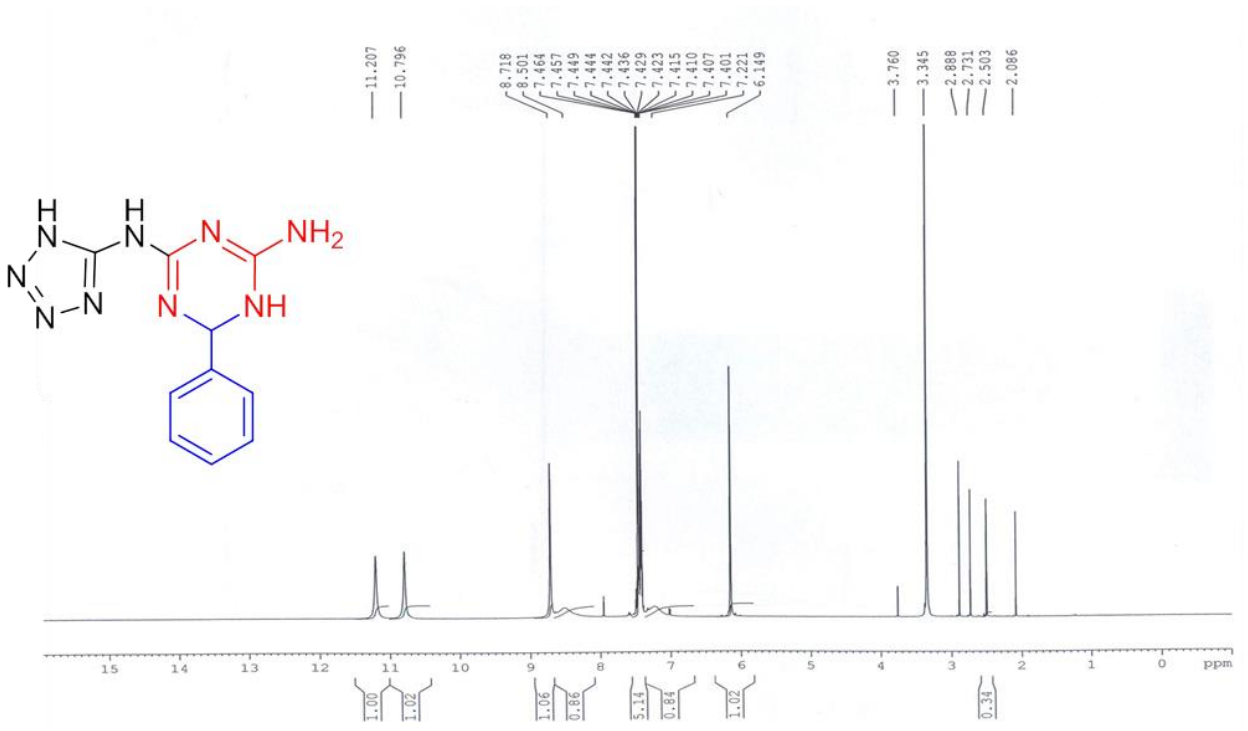

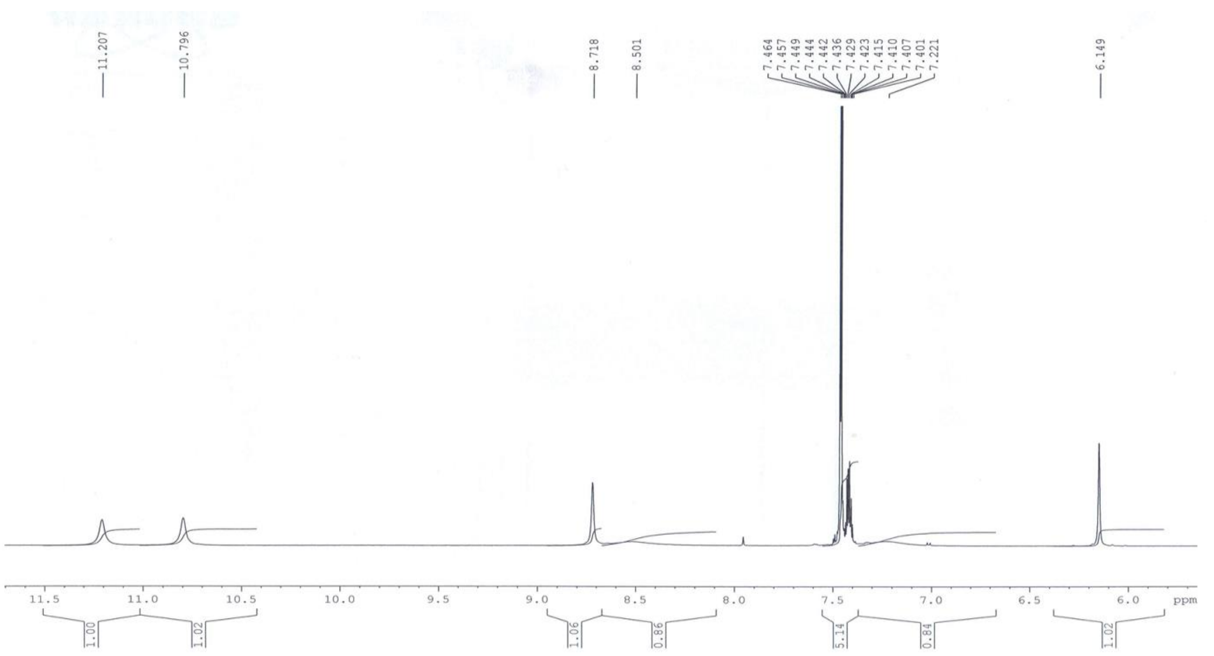

4c ^{13}C NMR spectrum (150 MHz, DMSO-d_6).

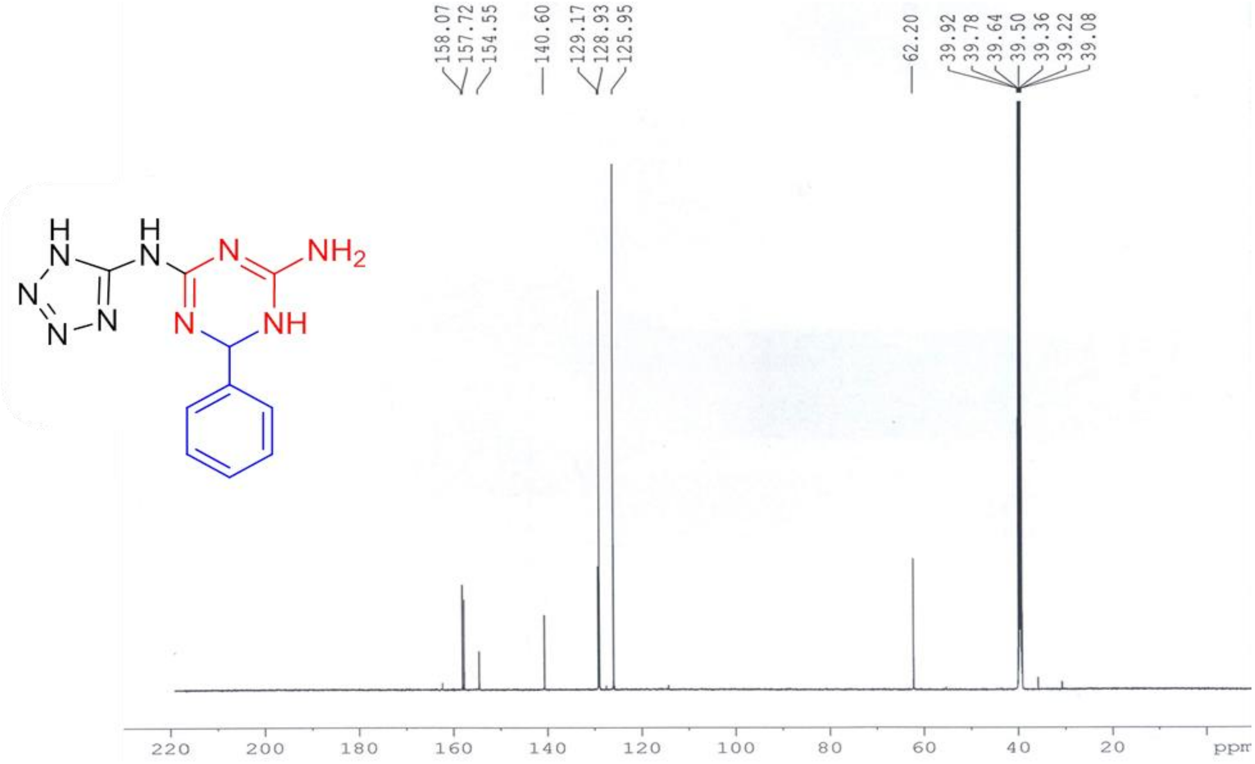

4c Mass spectrum.

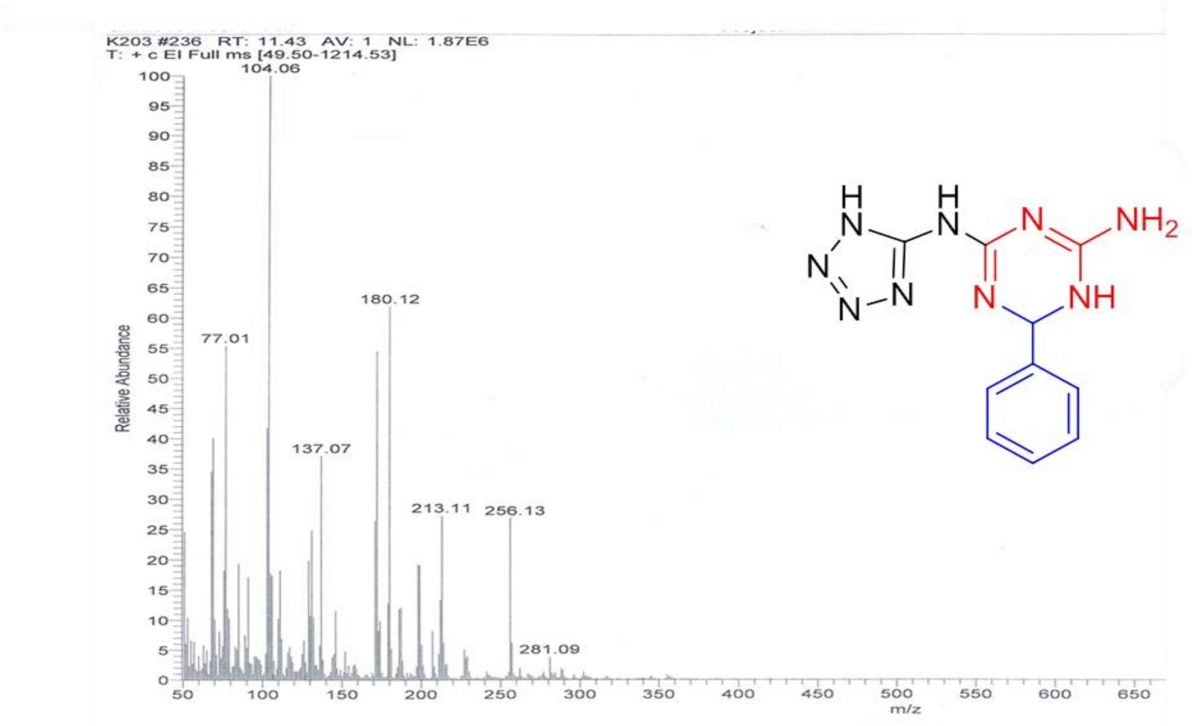

6-(Benzo[*d*][1,3]dioxol-5-yl)-*N*²-(5*H*-tetrazol-5-yl)-5,6-dihydro-1,3,5-triazine-2,4-diamine (4d).

This compound is novel.

4d ^{1}H NMR spectrum (600 MHz, DMSO-d_6).

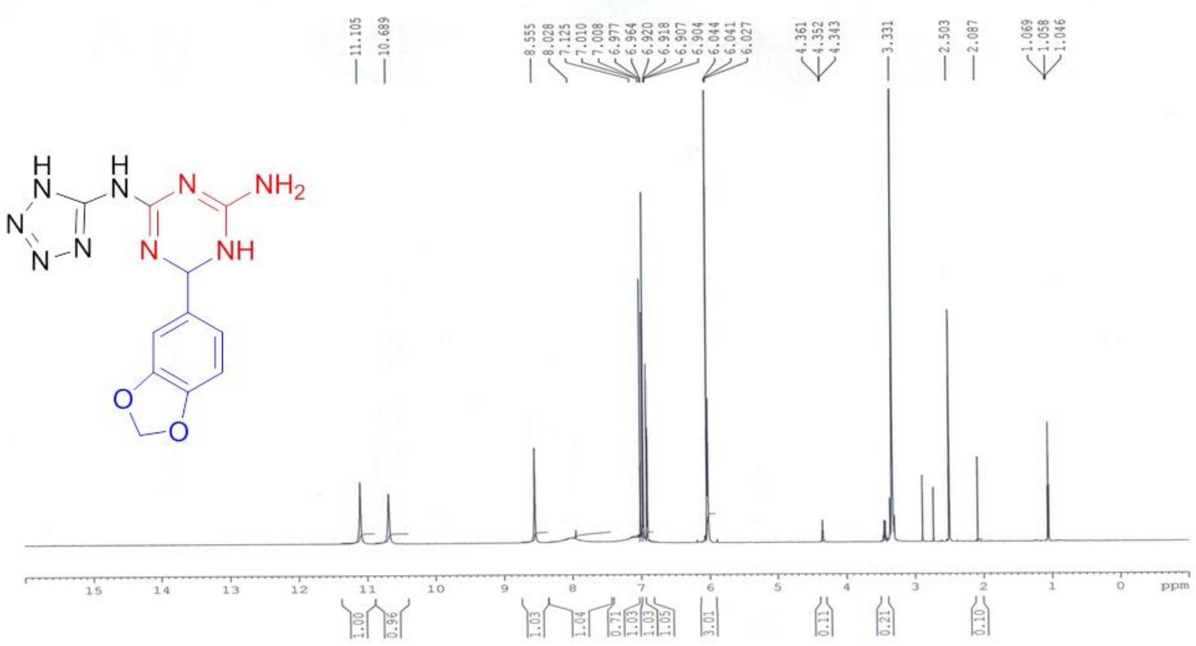

4d ^{13}C NMR spectrum (150 MHz, DMSO-d_6).

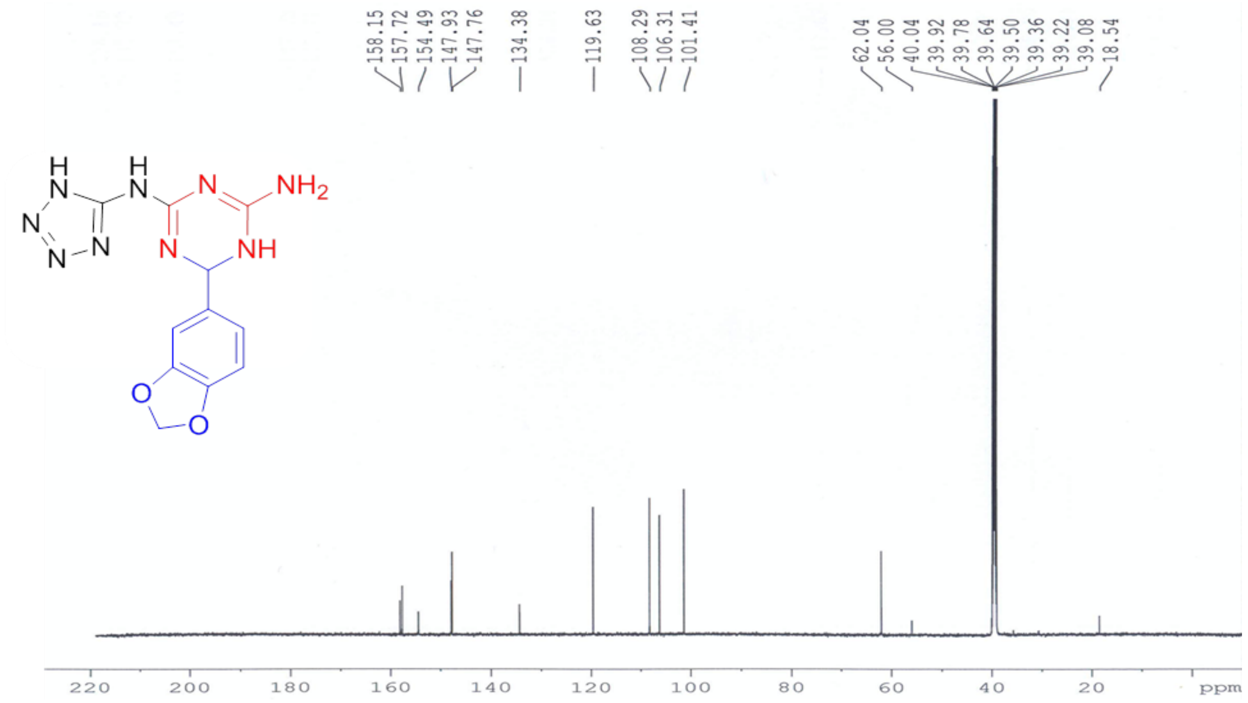

6-(2-Methoxyphenyl)-*N*²-(5*H*-tetrazol-5-yl)-5,6-dihydro-1,3,5-triazine-2,4-diamine (4e).

This compound is novel.

4e ¹H NMR spectrum (600 MHz, DMSO-d_6).

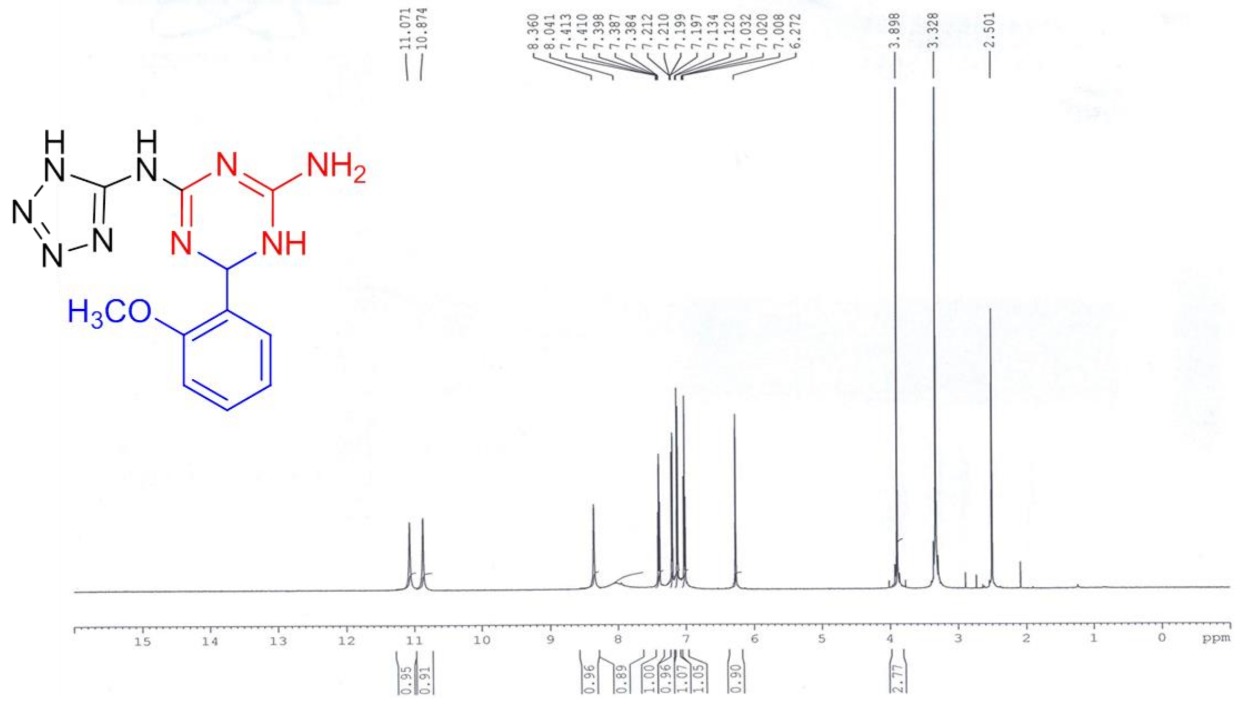

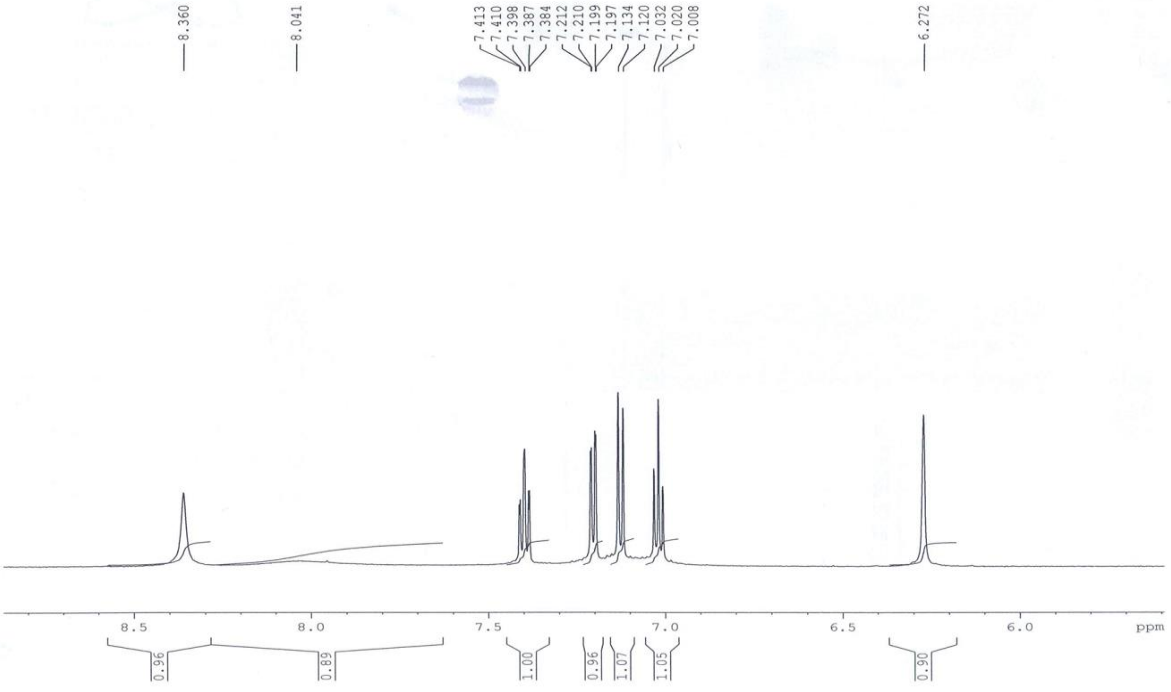

4e ^{13}C NMR spectrum (150 MHz, DMSO-d_6).

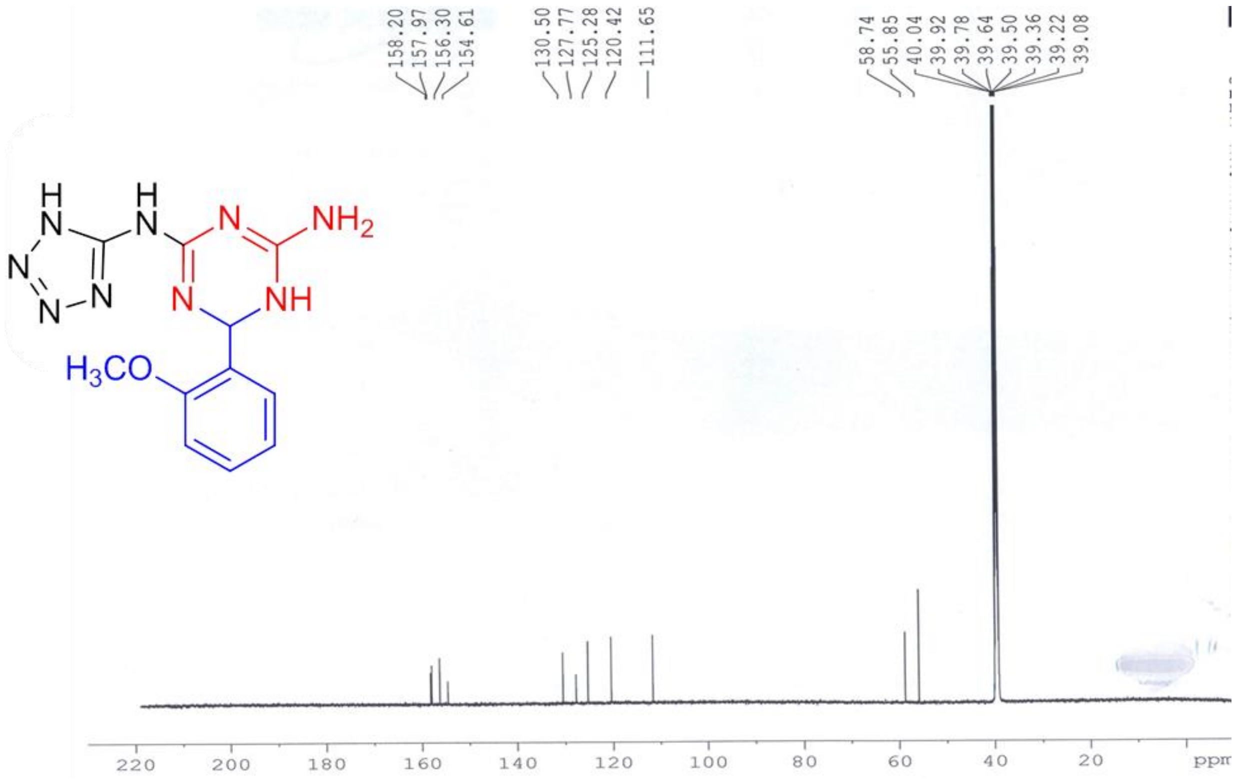

4e Mass spectrum.

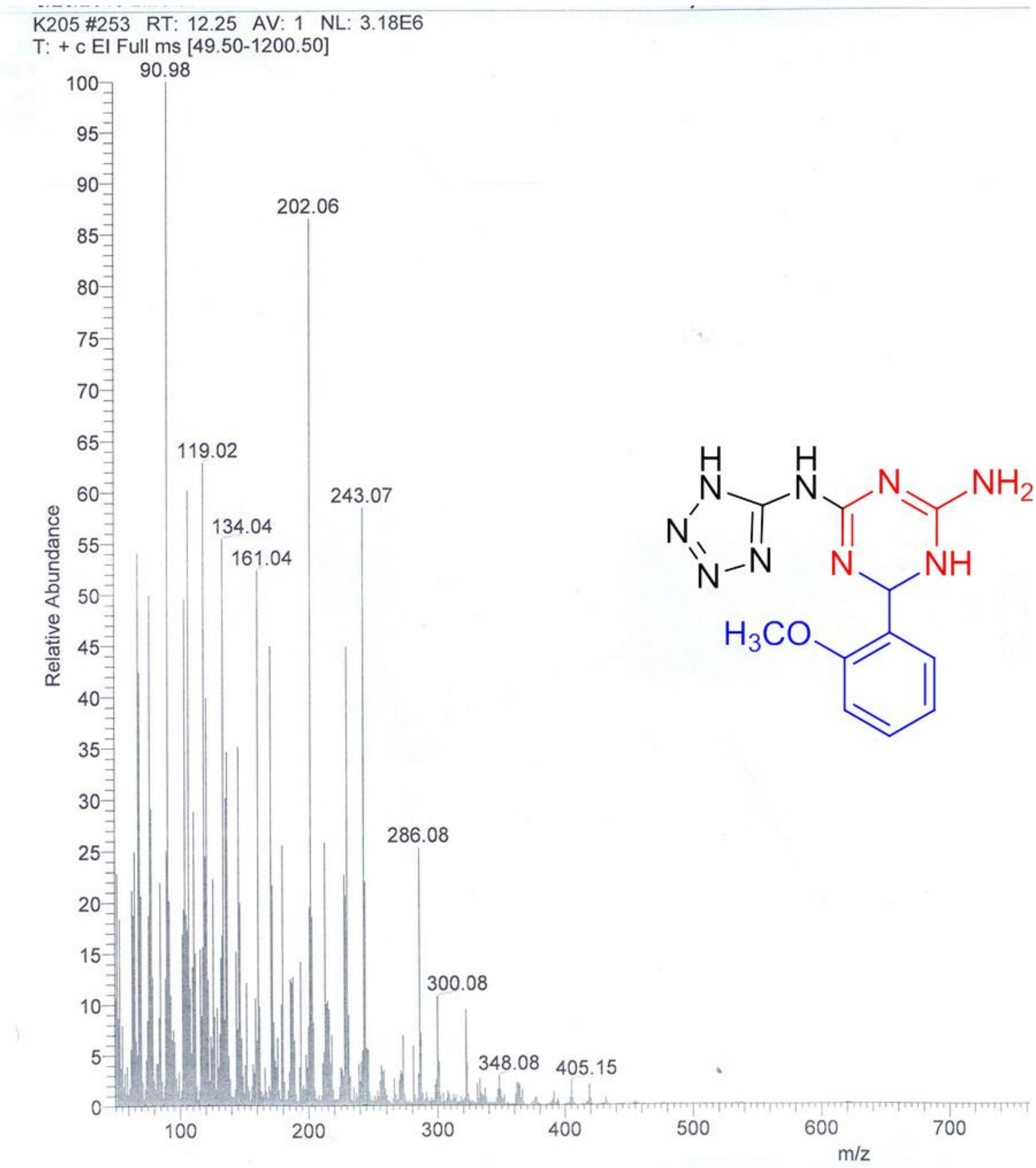

6-(4-Methylphenyl)-*N*^{2}-(5*H*-tetrazol-5-yl)-5,6-dihydro-1,3,5-triazine-2,4-diamine (4f).

This compound is novel.

4f ^{1}H NMR spectrum (600 MHz, DMSO-d_6).

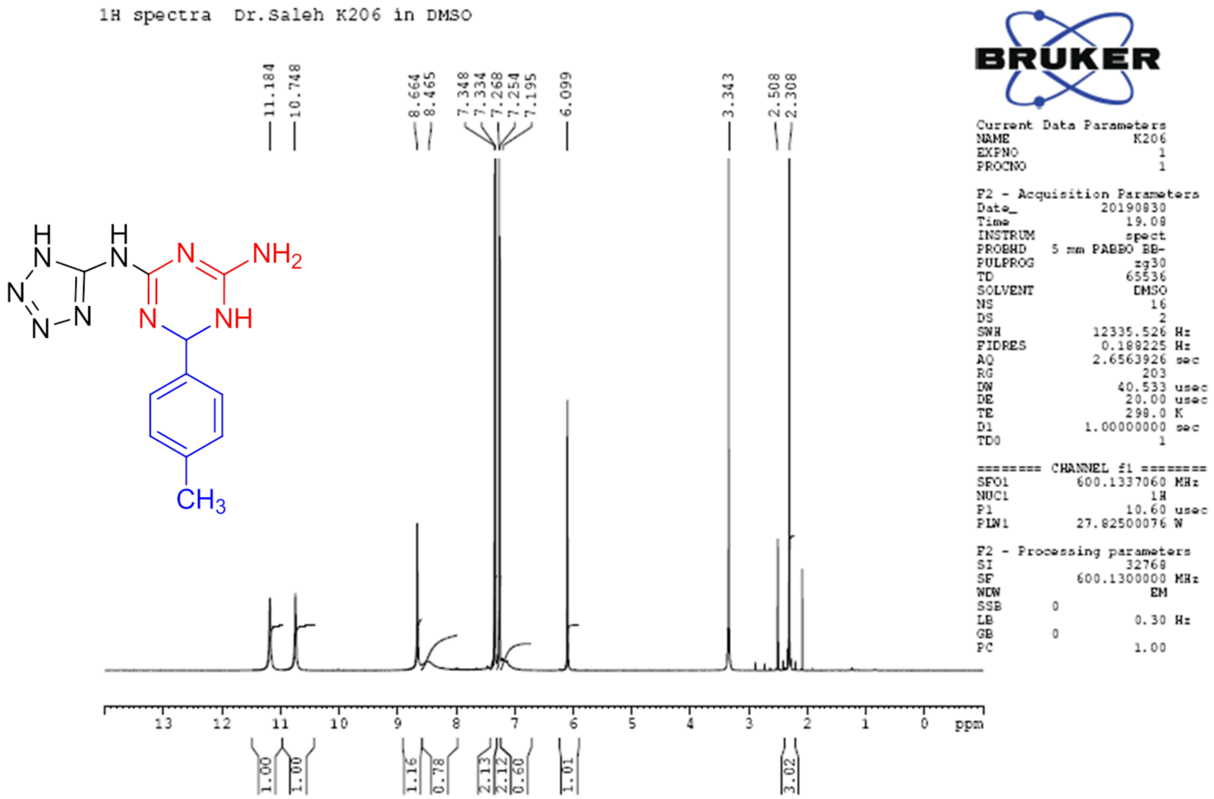

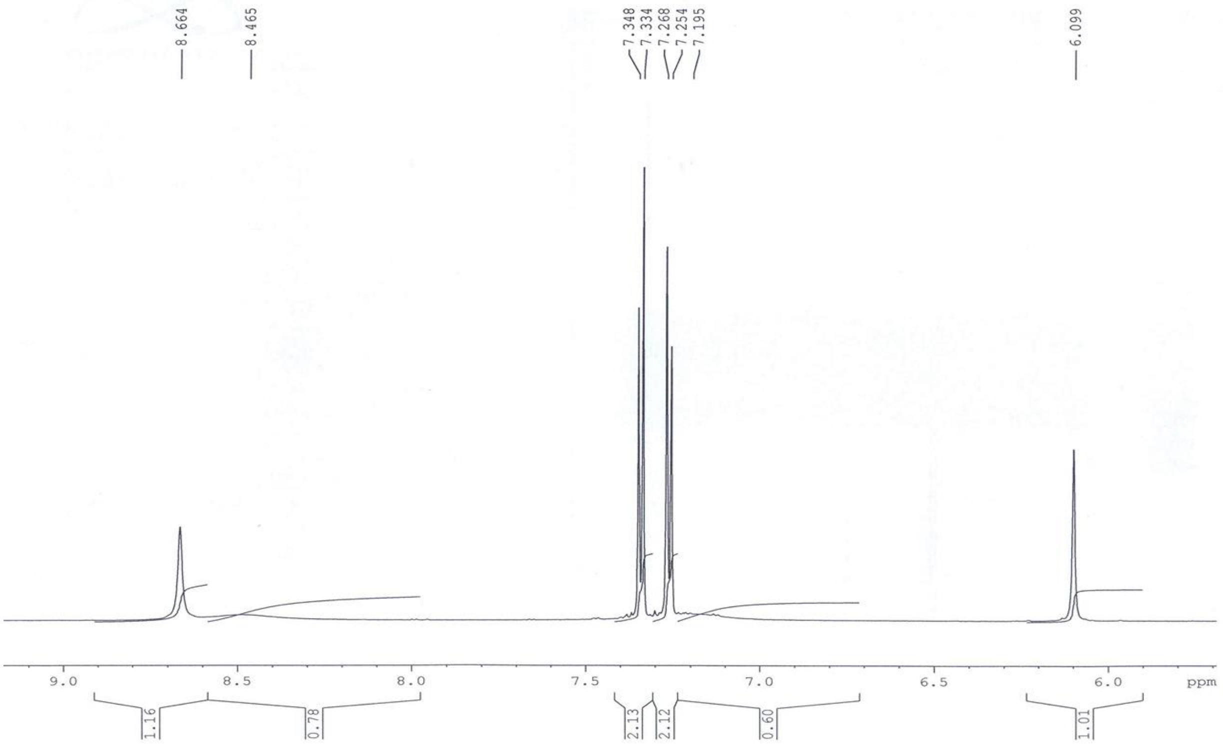

4f ^{13}C NMR spectrum (150 MHz, DMSO-d_6).

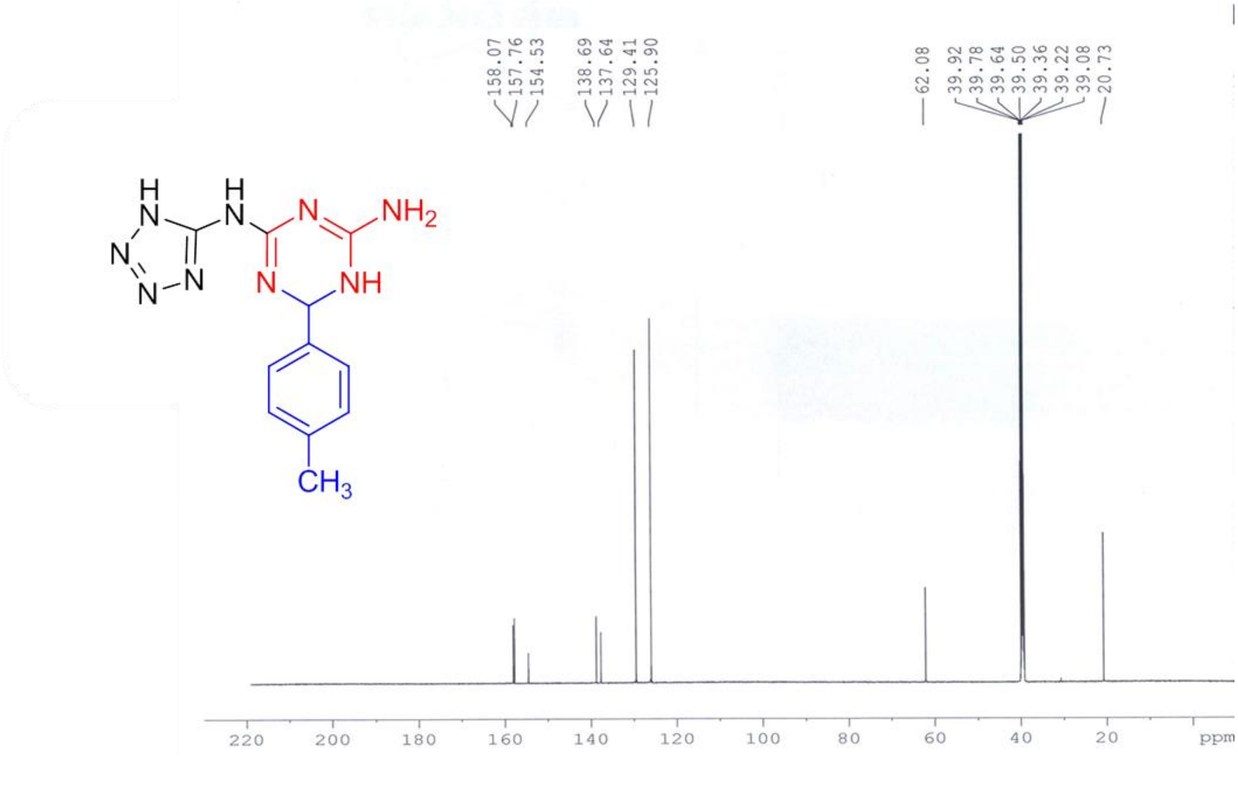

6-(2-Chlorophenyl)-*N*[2]-(5*H*-tetrazol-5-yl)-5,6-dihydro-1,3,5-triazine-2,4-diamine (4g).

This compound is novel.

4g ^{1}H NMR spectrum (600 MHz, DMSO-d_6).

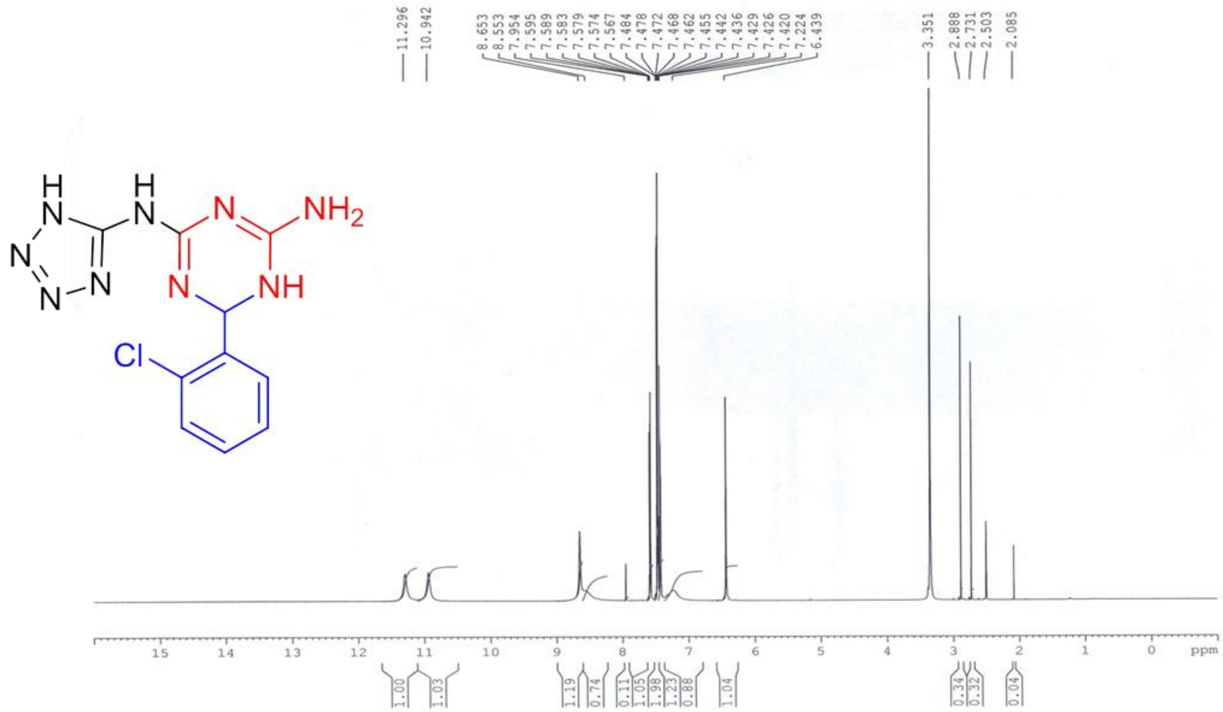

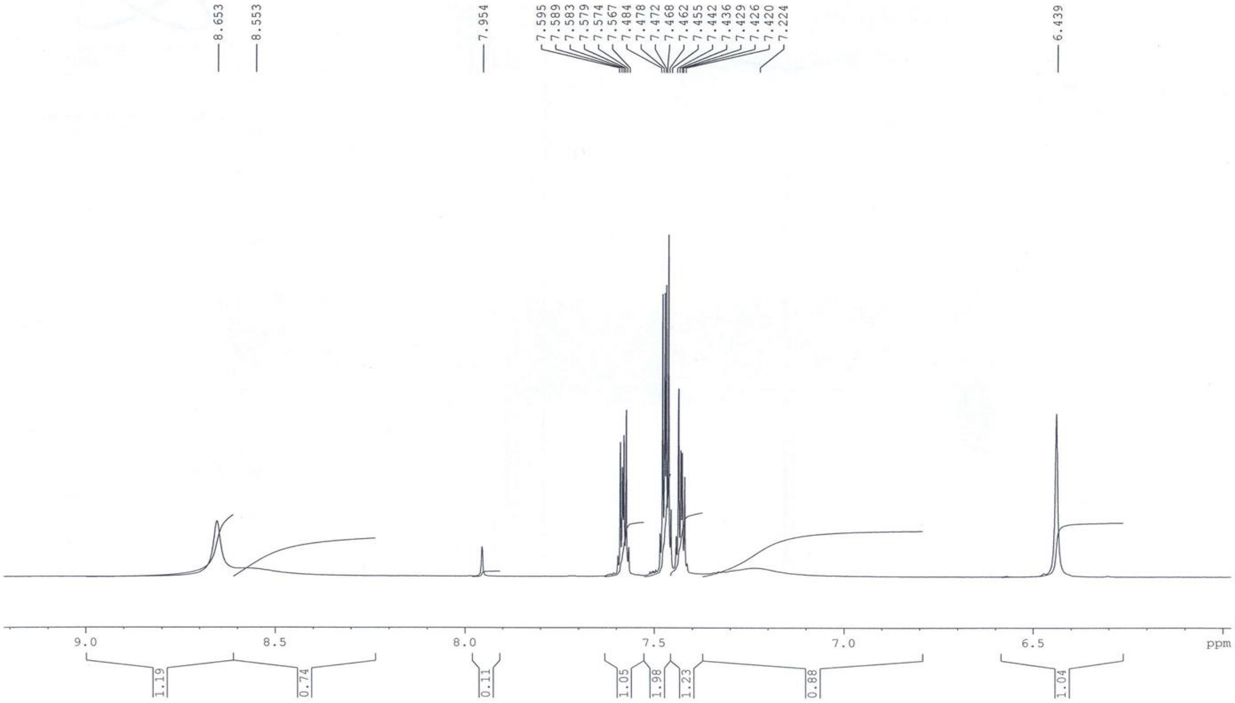

4g ^{13}C NMR spectrum (150 MHz, DMSO-d_6).

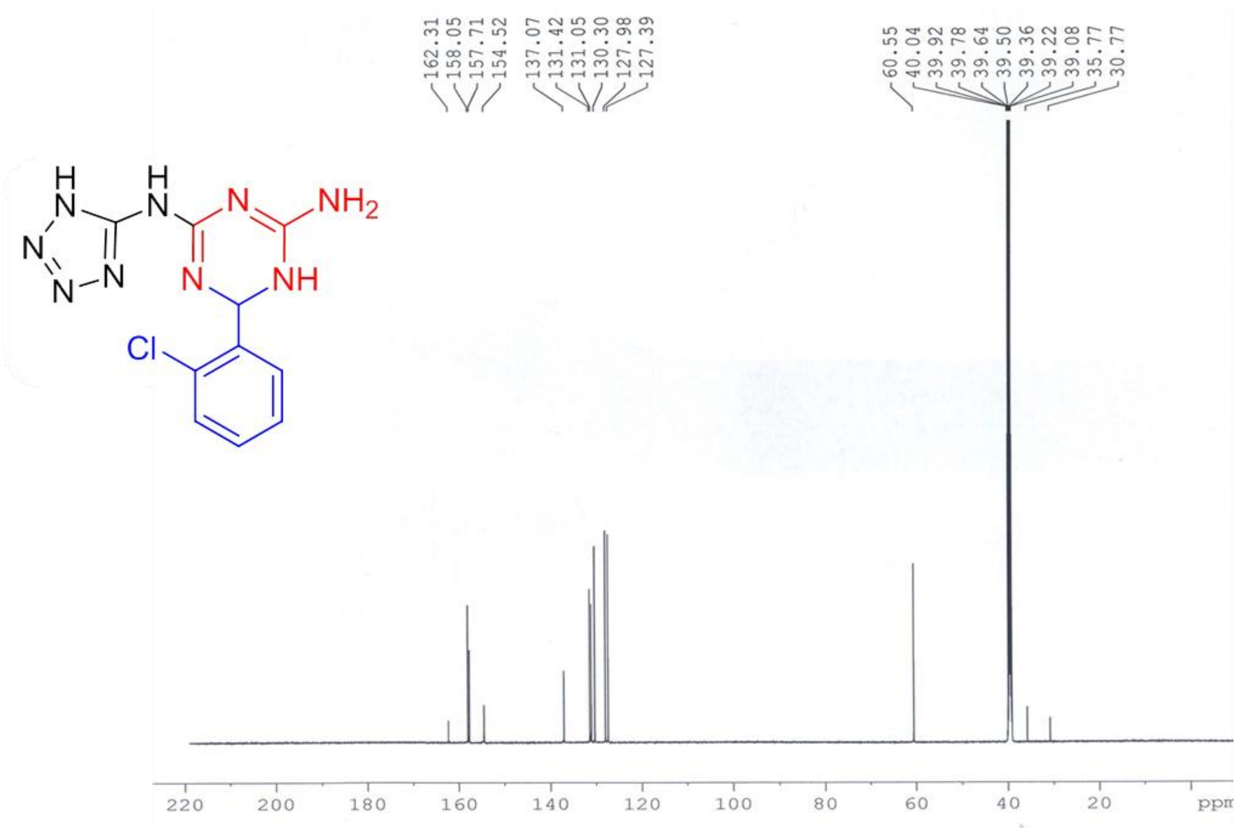

6-(3-Nitrophenyl)-*N*²-(5*H*-tetrazol-5-yl)-5,6-dihydro-1,3,5-triazine-2,4-diamine (4h).

This compound is novel.

4h ^{1}H NMR spectrum (600 MHz, DMSO-d_6).

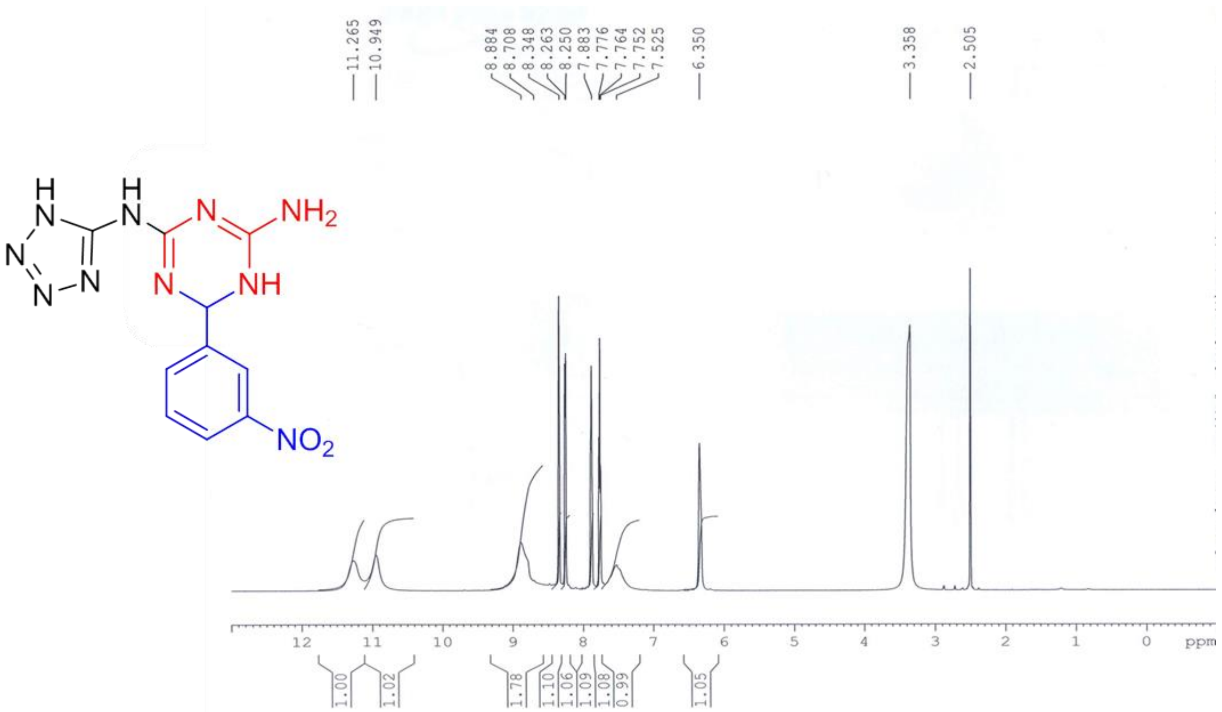

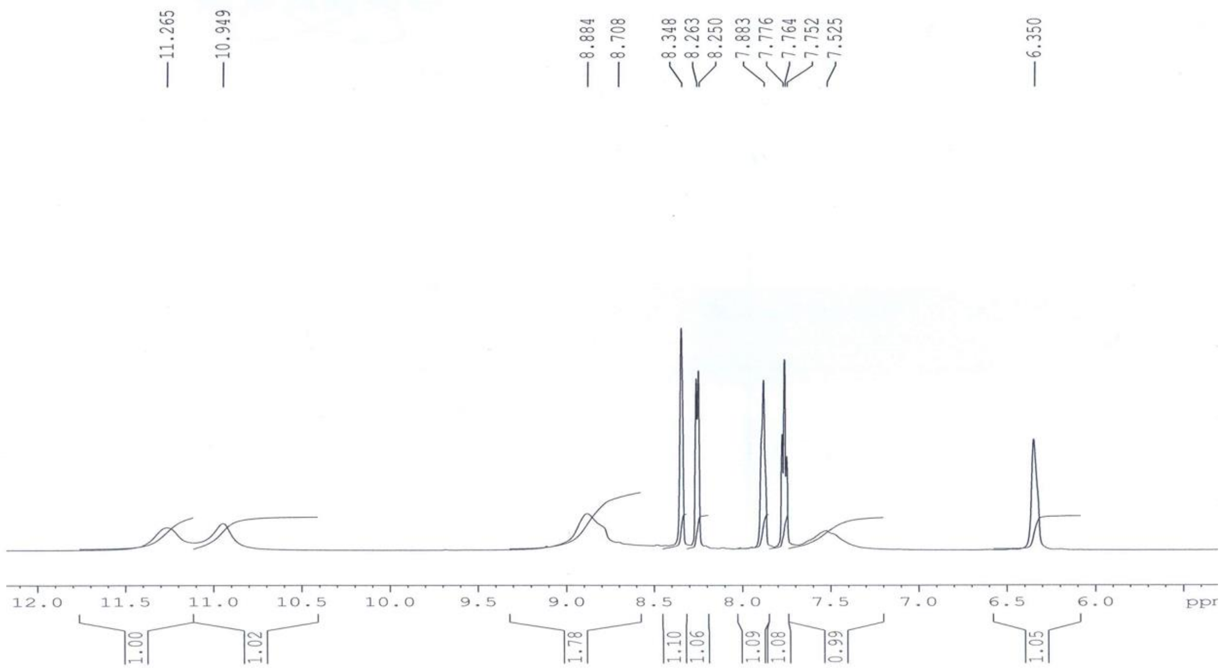

4h ^{13}C NMR spectrum (150 MHz, DMSO-d_6).

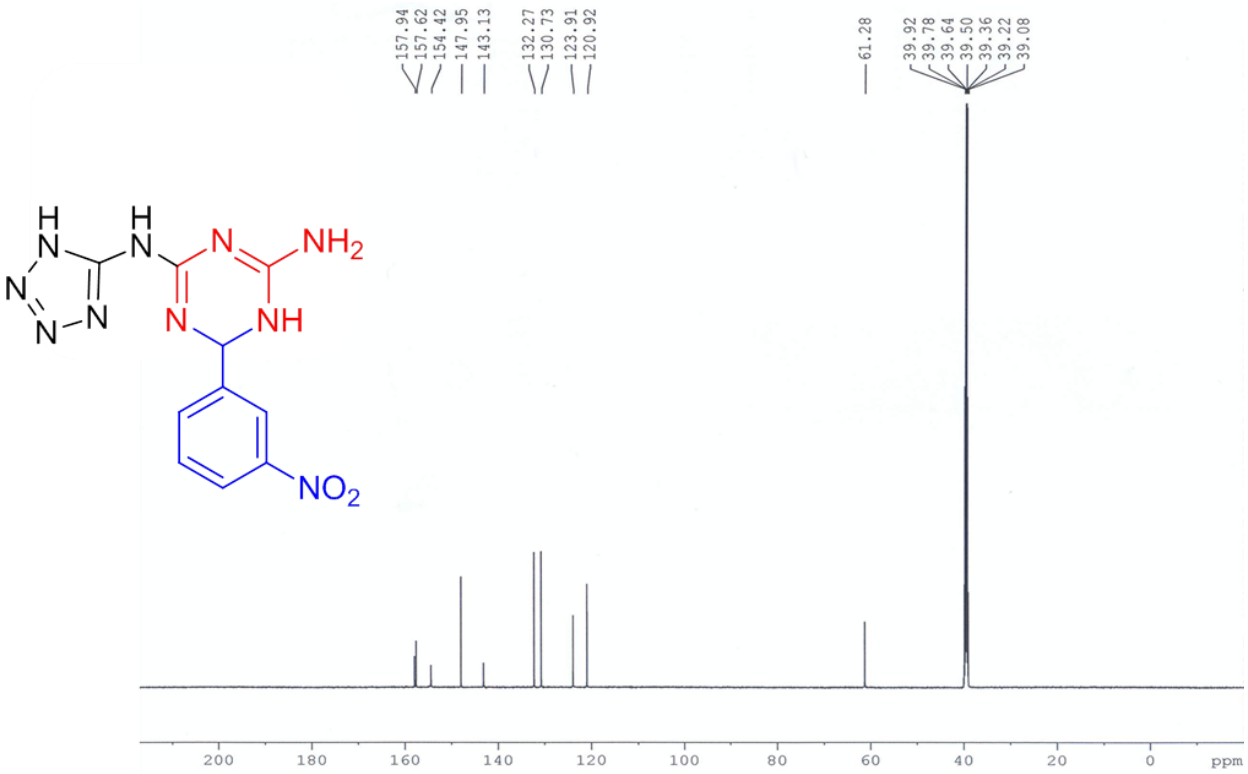

6-(Furan-2-yl)-*N*^{2}-(5*H*-tetrazol-5-yl)-5,6-dihydro-1,3,5-triazine-2,4-diamine (4i).

This compound is novel.

4i ^{1}H NMR spectrum (600 MHz, DMSO-d_6).

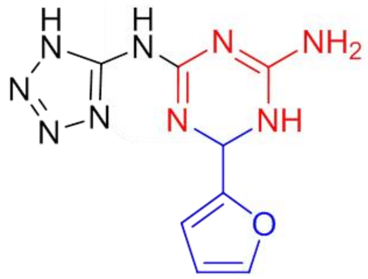

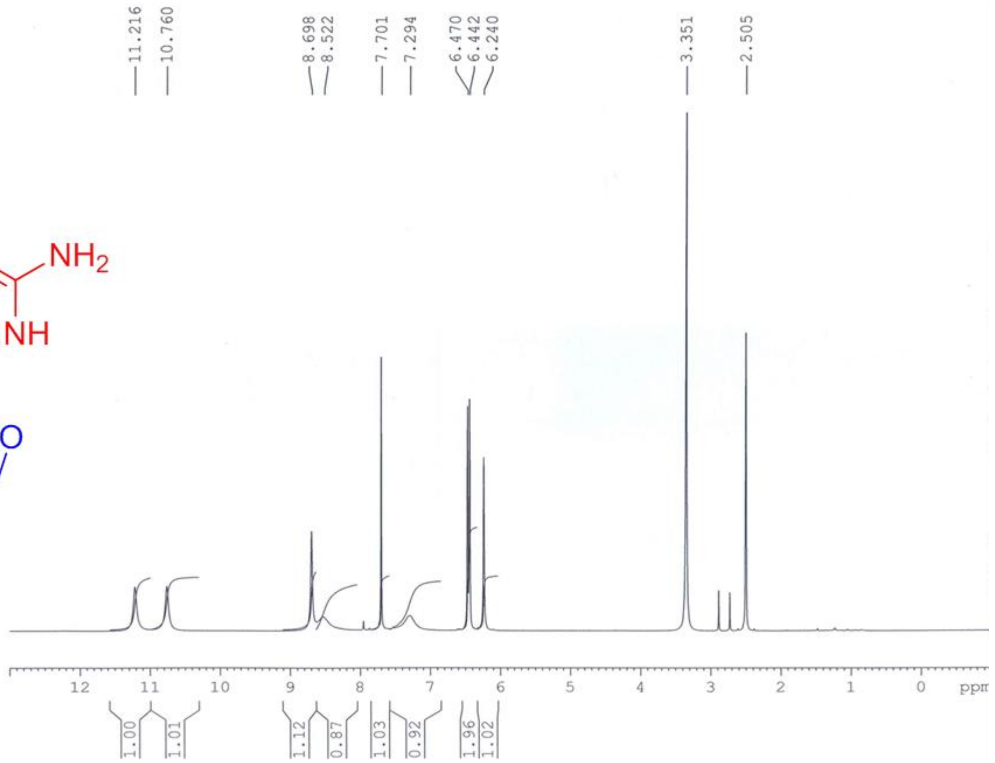

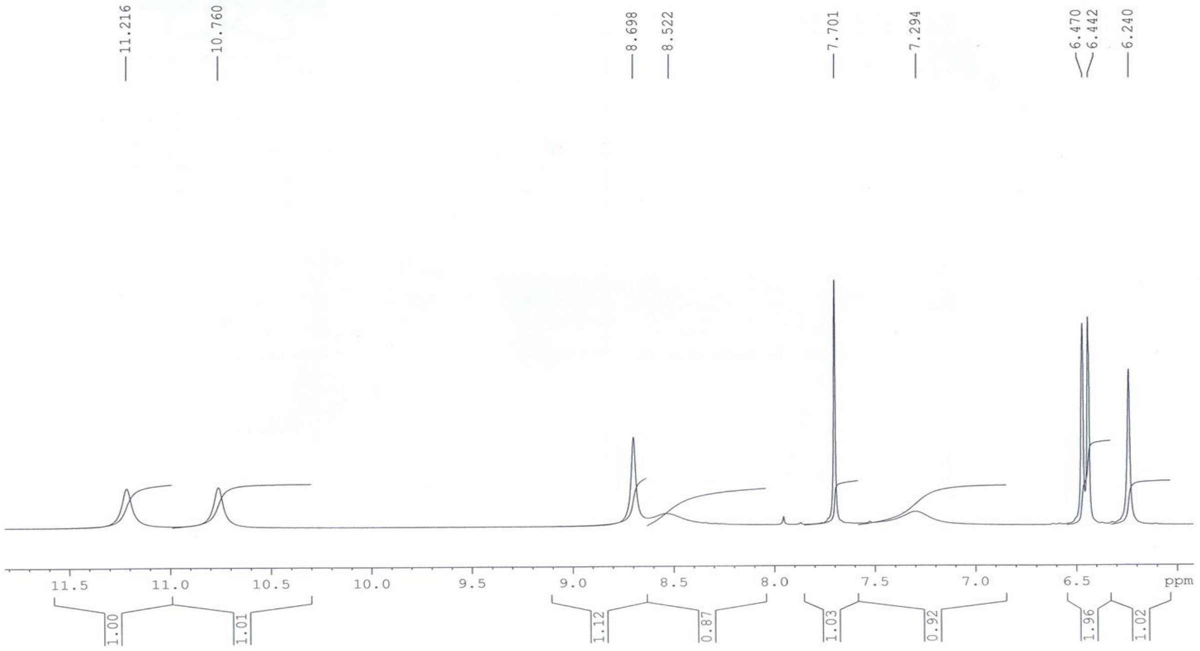

4i ^{13}C NMR spectrum (150 MHz, DMSO-d_6).

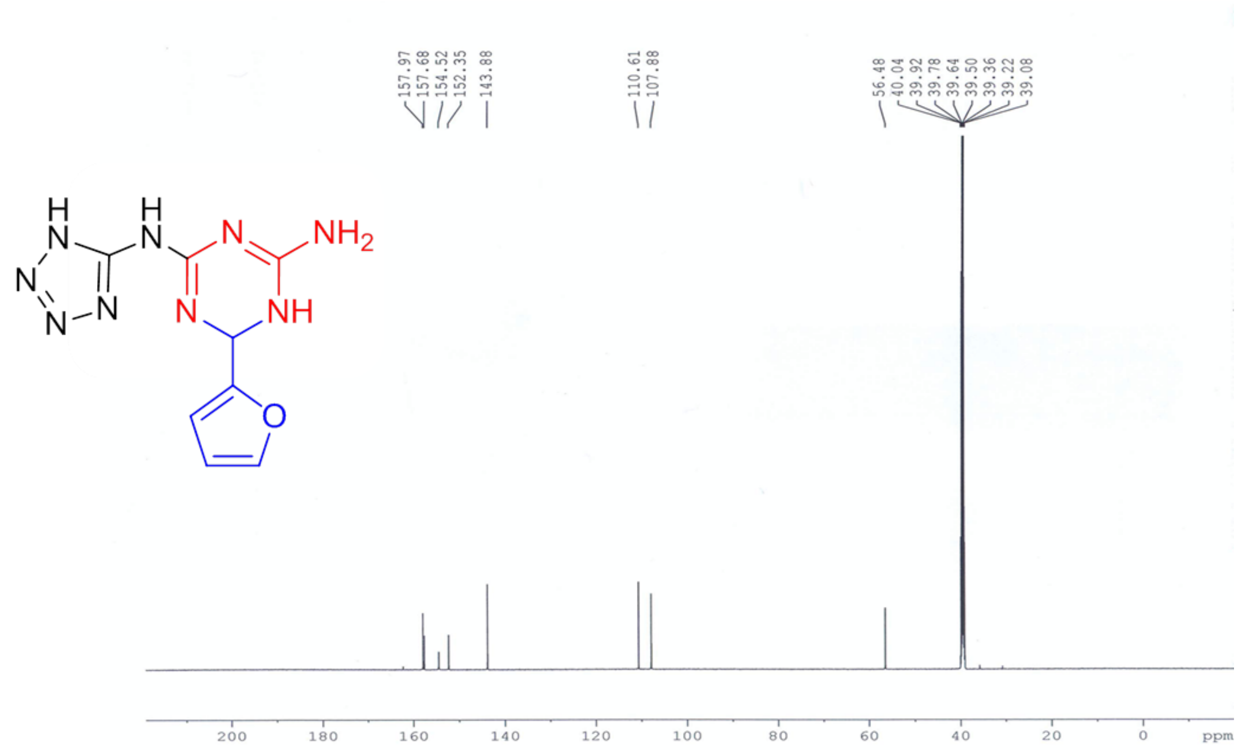

4i Mass spectrum

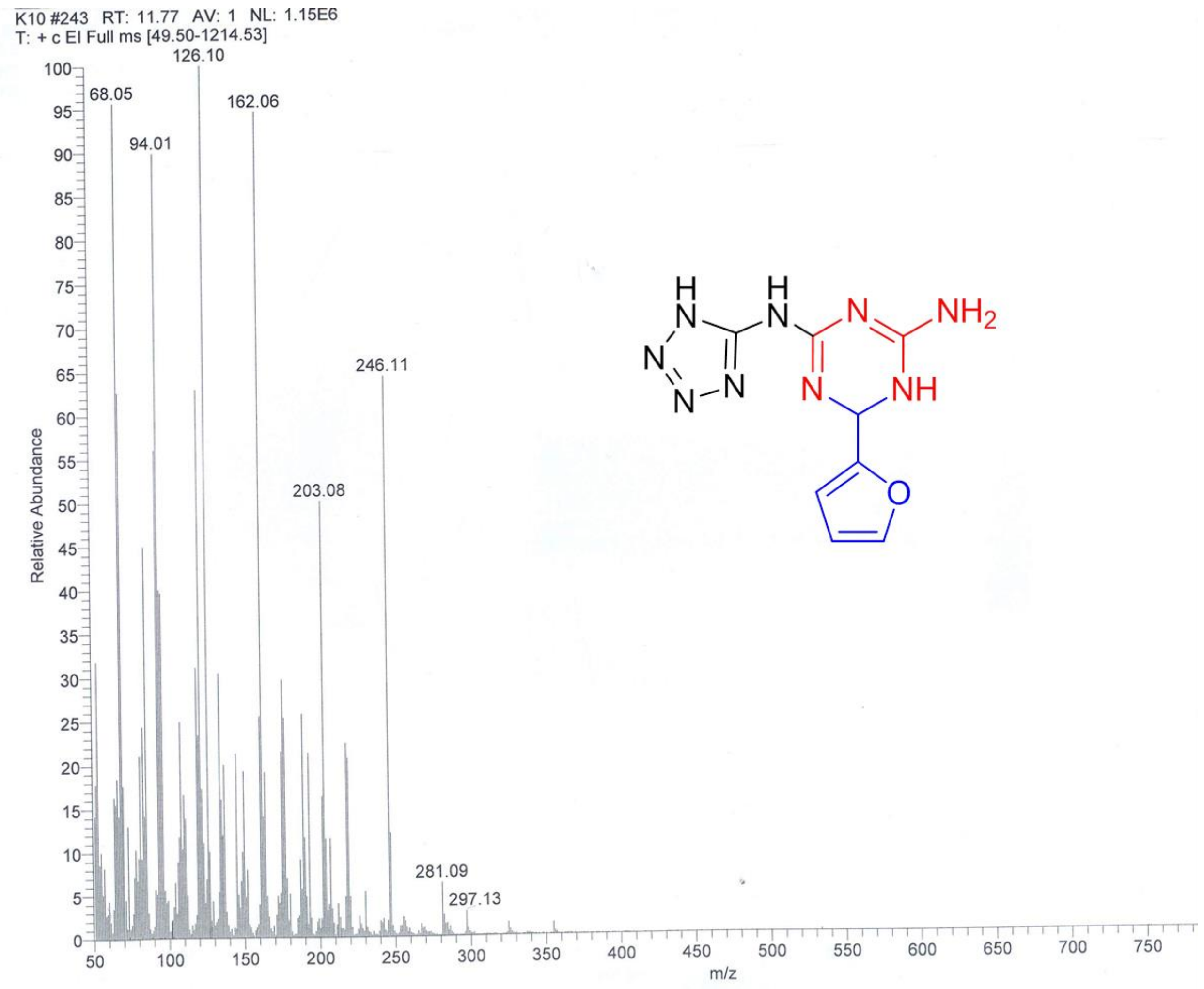

6-(4-*N*,*N*-Dimethylaminophenyl)-*N*^{2}-(5*H*-tetrazol-5-yl)-5,6-dihydro-1,3,5-triazine-2,4-diamine (4j).

This compound is novel.

4j ^{1}H NMR spectrum (600 MHz, DMSO-d_6).

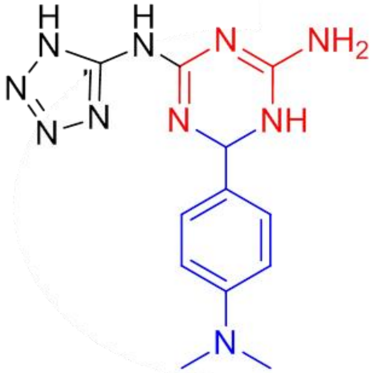

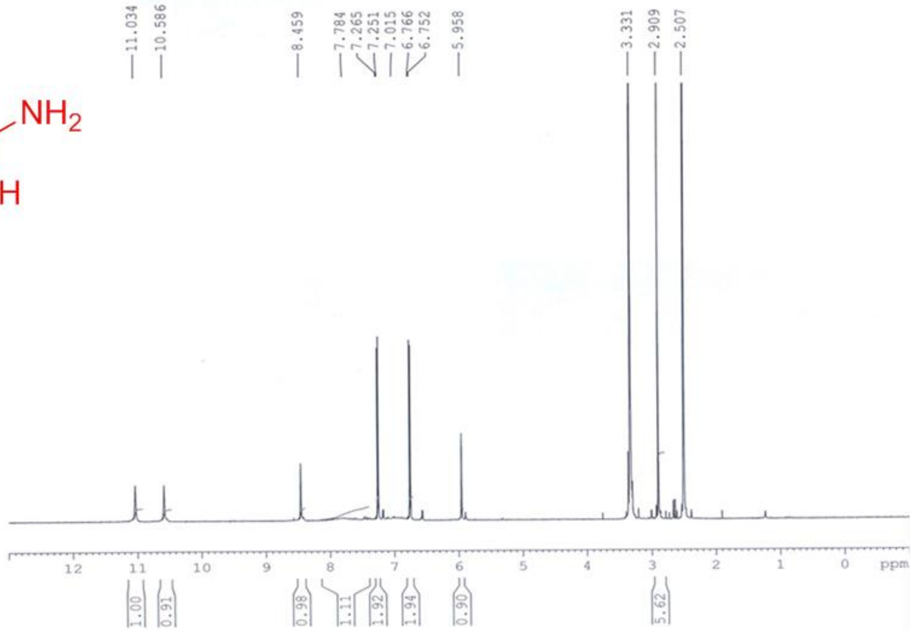

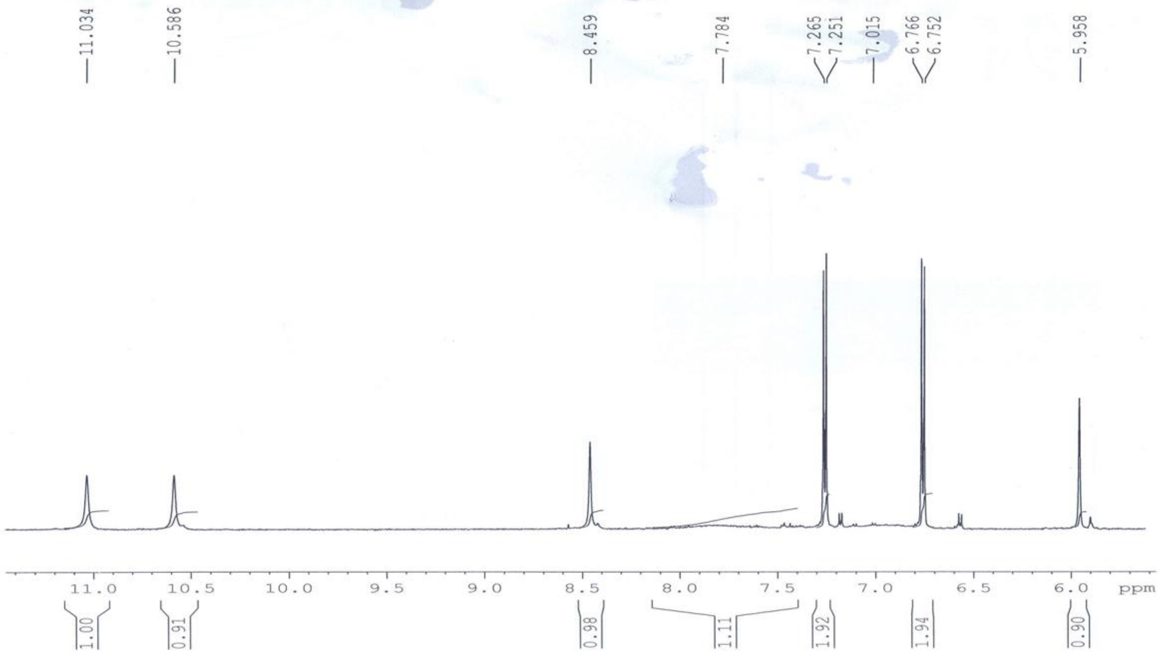

4j ^{13}C NMR spectrum (150 MHz, DMSO-d_6).

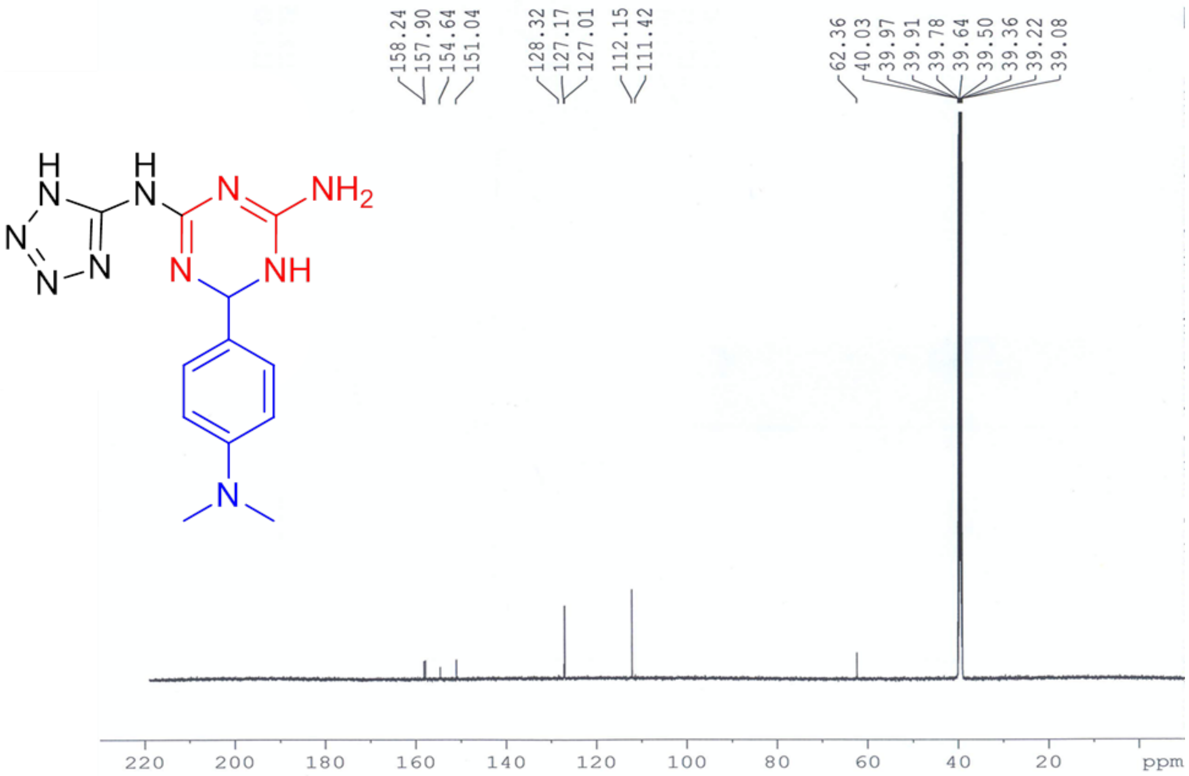

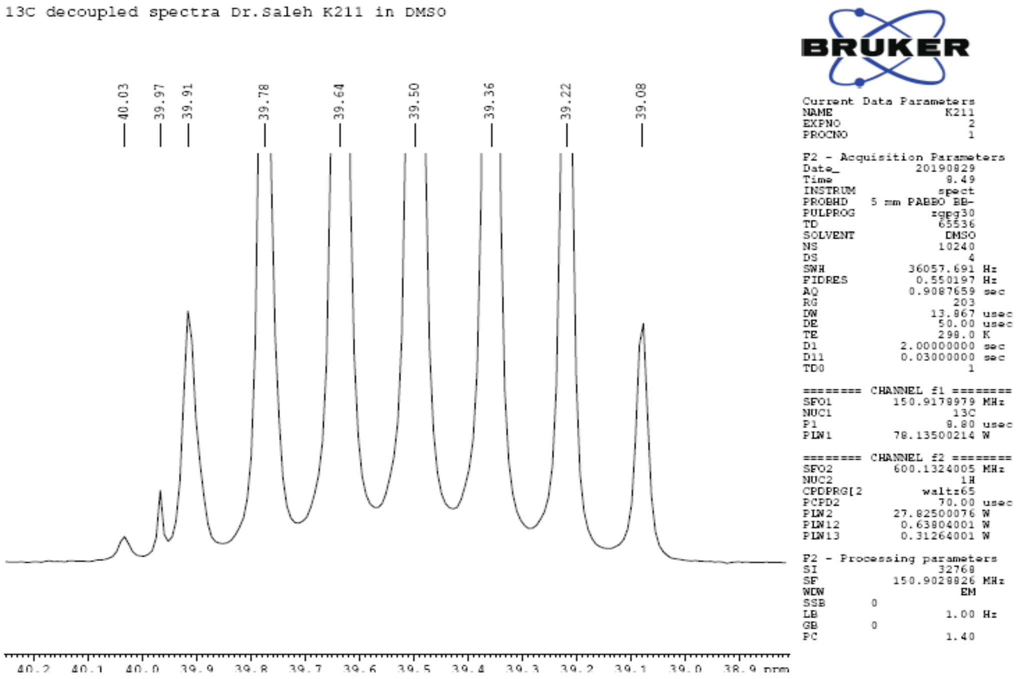

13C decoupled spectra Dr.Saleh K211 in DMSO

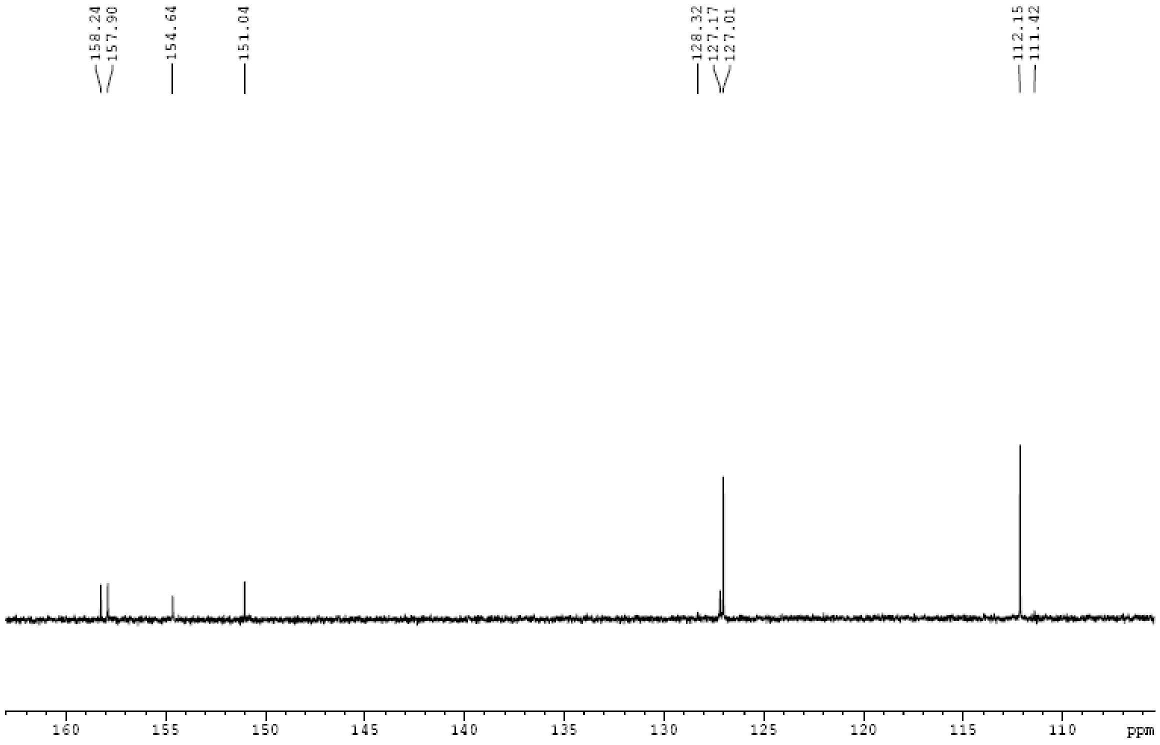

Supplement: File 1 — NMR and mass spectra. [file Beilstein_J_Org_Chem-16-1706-s001.pdf]
